# Connecting Thermodynamics and Kinetics of Proton Coupled Electron Transfer at Polyoxovanadate Surfaces Using the Marcus Cross Relation

**DOI:** 10.1021/acs.inorgchem.2c02541

**Published:** 2022-09-01

**Authors:** Alex A. Fertig, Ellen M. Matson

**Affiliations:** Department of Chemistry, University of Rochester, Rochester, New York 14627, United States

## Abstract

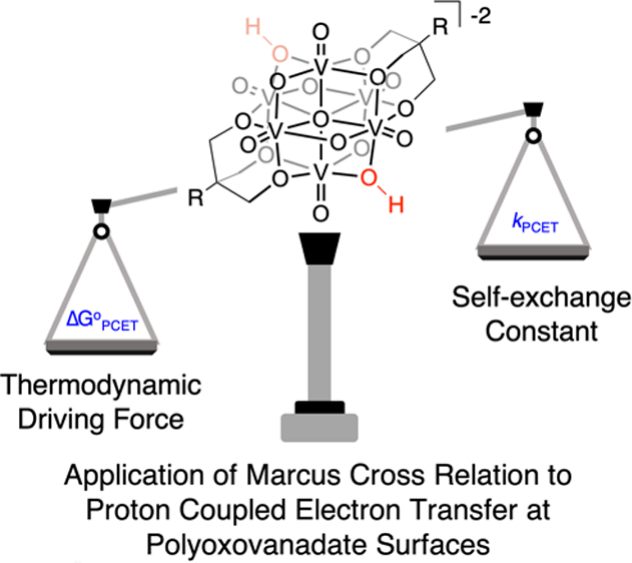

Here, we evaluate the efficacy of multiple methods for elucidating the average bond dissociation free energy (BDFE) of two surface hydroxide moieties in a reduced polyoxovanadate cluster, [V_6_O_11_(OH)_2_(TRIOL^NO2^)_2_]^−2^. Through cyclic voltammetry, individual thermochemical parameters describing proton coupled electron transfer (PCET) are obtained, without the need for synthetic isolation of intermediates. Further, we demonstrate that a method involving a series of open circuit potential measurements with varying ratios of reduced to oxidized clusters is most attractive for the direct measurement of BDFE(O-H) for polyoxovanadate clusters as this approach also determines the stoichiometry of PCET. We subsequently connect the driving force of PCET to the rate constant for the transfer of hydrogen atoms to a series of organic substrates through the Marcus cross relation. We show that this method is applicable for the prediction of reaction rates for multielectron/multiproton transfer reactions, extending the findings from previous work focused on single electron/proton reactions.

## Abstract 2

The thermochemical parameters of proton coupled electron transfer at the surface of a polyoxovanadate cluster are established through multiple electrochemical methods. From these values, the driving force of hydrogen atom transfer to a series of organic substrates can be calculated and used to predict the rate of reaction through the Marcus cross relation.

## Introduction

The uptake of hydrogen atoms (H atoms) in metal oxides is an important chemical reaction, with relevance to emerging energy storage and conversion technologies.^1−3^ The formal transfer of H atom equivalents (i.e., the movement of protons and electrons) can occur by several mechanisms (e.g., electron-proton co-doping, H atom spill over).^4−6^ While there has been a recent increase in interest in the analysis of proton coupled electron transfer in extended metal oxide structures,^7,8^ the development of an in-depth understanding as to how these reactions occur is complicated by hydrogen intercalation and the existence of defect sites at the surface of heterogeneous metal oxides. Limited approaches for *in situ* analysis of the surfaces of bulk materials during H atom transfer (HAT) further obfuscates the elucidation of PCET reactivity at the atomic level. Accordingly, alternative methods to advance the understanding of proton coupled electron transfer (PCET) at metal oxide interfaces are needed.

One approach to circumvent challenges associated with the analysis of interfacial PCET involves the study of dimensionally reduced analogues of metal oxide materials. Molecular metal oxide assemblies well-suited for these investigations are polyoxometalates (POMs). These clusters are composed of transition-metal oxyanions, bound together by bridging oxide ligands. The resultant three-dimensional structures resemble the surface morphology of heterogeneous metal oxide materials, with alternating bridging and terminal oxide ligands. POMs possess significant solubility in organic and aqueous solvent, facilitating the *in situ* analysis via well-established analytical techniques reserved for molecular species.

Many research groups, including our own,^10−14^ have leveraged the distinct physicochemical properties of POMs to understand charge transfer and compensation at metal oxide interfaces.^15−19^ Electrochemical analysis of POMs has shown that a dependency on the ability to perform electron transfer is directly related to the presence and identity of charge compensating species (e.g., alkali metals, protons). In particular, the anodic shift in potential required for reduction observed upon acidification of the system is characteristic of PCET as coupling protonation to electron transfer results in a decrease in the energy required for the charge transfer process to occur. Similar pH-dependent shifts in reduction potentials have been shown in metal oxides,^20−22^ where PCET has been demonstrated as a functional mode of surface reactivity. Notably, the reactivity of reduced POMs is limited to the surface of the cluster, eliminating the intercalation pathway available to extended solids.

To date, the majority of work focused on charge compensation at POM surfaces has been rooted in their application in charge storage devices,^23−25^ with in-depth evaluation of the thermochemistry of H atom uptake largely overlooked. Indeed, there are only two examples of thermochemical studies quantifying the bond dissociation free energy (BDFE) of H atoms at the surface of reduced POMs. Sami and coworkers measured the BDFE (O-H) of the resulting hydroxide ligand formed upon reduction of a vanadium-doped polyoxotungstate cluster, [HPV_2_W_10_O_40_]^−5^.^26^ Subsequently, our group reported the average bond strength of bridging hydroxide ligands at a reduced polyoxovanadate-alkoxide (POV-alkoxide) cluster, [V_6_O_7_ (OH)_6_(TRIOL^NO2^)_2_]^−2^, ultimately extending the reactivity of hydrogen equivalents at the surface of a POM to small molecule activation.^27^

The limited evaluation of O–H bond strengths at reduced POM surfaces can be attributed to the fact that traditional approaches for determination of BDFEs require the isolation of intermediates at vertices of a square scheme. Reduced and protonated POMs are prone to disproportionation, rendering direct measurement of p*K*a and redox potential challenging. Recent advances in the field of PCET have demonstrated several methods for measuring BDFE (E-H) (E = O, N, C, etc.) values that alleviate the necessity for isolating reactive intermediates, rendering the quantification of the BDFE(O-H) of surface hydroxide moieties in reduced POMs ripe for re-evaluation. For example, translation of the concepts of Pourbaix diagrams into organic solvent has been accomplished by measuring the electrochemical response of a compound upon the introduction of a series of organic acids with varying strengths.^28,29^ More recently, new methodology for the evaluation of the thermochemistry of PCET has been achieved through open circuit potential measurements.^30^ In this approach, varying the relative concentration of reduced to oxidized species and measuring the energy of the system allow for the direct measurement of the BDFE(E-H).

While analysis of the free energy of PCET has been demonstrated for many molecular donor and acceptor pairs, there remain a few examples that draw a connection between the thermodynamic driving force and rates of reaction of HAT. In a series of reports, Mayer has correlated reaction rate and driving force using a modified version of the Marcus cross relation; the rate of reaction can be calculated using the thermochemical driving force and the H atom self-exchange constants for each reagent.^31−33^ Calculated rates for HAT reactions were in good agreement with experimental results (within one or two orders of magnitude). While there have been no examples in which rate constants of PCET from a POM have been predicted using the Marcus cross relation, work from Sami and coworkers used the cross relation to predict the self-exchange constant of H atoms at the metal oxide surface.^26^ This finding suggests that the Marcus cross relation can be applied to accurately predict the rate of HAT reactions at metal oxide surfaces.

Here, we report the BDFE(O-H)_avg_ of hydroxide moieties at the surface of a POV-alkoxide cluster, [V_6_O_13_(TRIOL^NO2^)_2_]^2–^ (**V_6_O_13_^–2^**; TRIOL^NO2^ = (OCH_2_)_3_CNO_2_). Relating the reduction potential of the cluster to the strength of acid present allows for the thermochemical parameters required for the transfer of protons and electrons to be established. Additionally, the free energy for H atom transfer is measured directly using open circuit potential analysis. With BDFE(O-H)_avg_ in hand, we demonstrate a relationship between the driving force for H atom transfer and rate of reaction, providing evidence for the efficacy in using Marcus theory. Collectively, this work evaluates techniques to accurately analyze the thermodynamics of PCET at the surface of POMs, in addition to establishing the connection between changes in free energy and the rate of reaction for PCET for multielectron/multiproton reactions.

## Results and Discussion

### Determination of Bond Dissociation Free Energy of Surface O-H Bonds in V_6_O_11_(OH)_2_^–2^

**Figure 1:**
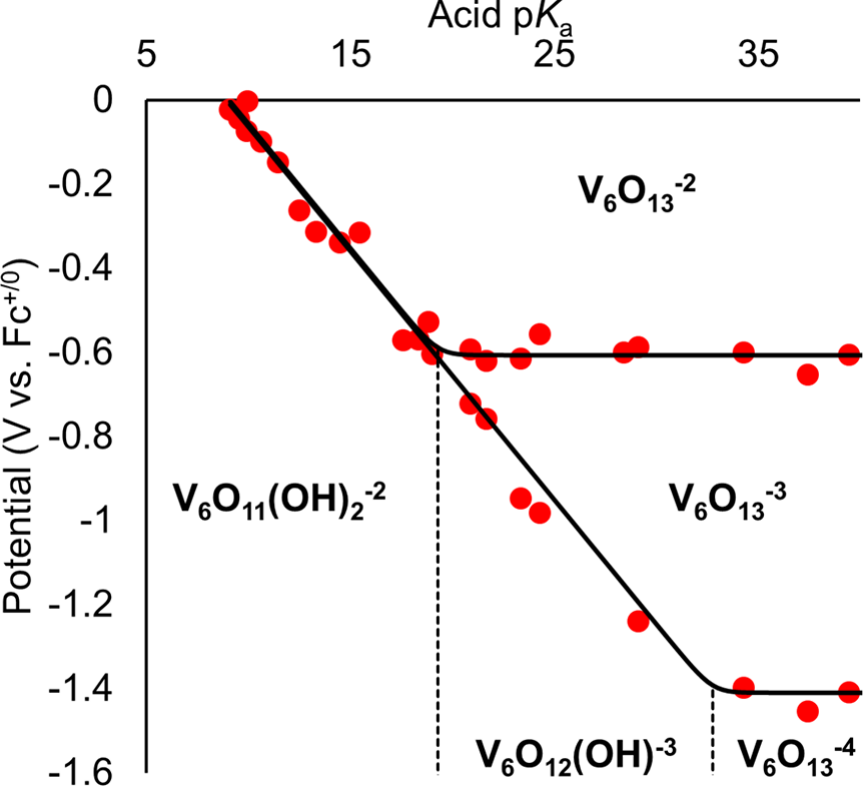
Potential–p*K*_a_ diagram for **V_6_O_13_^–2^**. Red data points represent the measured reduction potential(s) of 1 mM of **V_6_O_13_^–2^** in acetonitrile in the presence of 2 mM of various organic acids and supporting electrolyte (0.1 M [^n^Bu_4_N][PF_6_]). Reduction potentials are plotted against the p*K*_a_ of the organic acid used in each experiment. The horizontal black lines represent the acid-independent redox events, and the diagonal line represents the acid-dependent redox events, with a fixed slope of 59 mV/dec anchored at a p*K*_a_ value of 32.7. See the SI for more information, and for the cyclic voltammogram associated with each data point (Figures S1–S22). Each region of the diagram is labeled with the most stable species, including **V_6_O_13_^–2^**, [V_6_O_11_(OH)_2_(TRIOL^NO2^)_2_]^−2^ (**V_6_O_11_(OH)_2_^–2^**), [V_6_O_13_(TRIOL^NO2^)_2_]^−3^ (**V_6_O_13_^–3^**), [V_6_O_13_(TRIOL^NO2^)_2_]^−4^ (**V_6_O_13_^–4^**), and [V_6_O_12_(OH)(TRIOL^NO2^)_2_]^−3^ (**V_6_O_12_(OH)^−3^**).

To develop a thermochemical understanding of PCET at the surface of POMs, we analyzed electrochemical properties of **V_6_O_13_^–2^** in acetonitrile in the presence of a series of organic acids with pKa values ranging from 9.1 to 39.5 (Table S1 and Figures S1–S22, *vide infra*).^9,34−38^ Discussion of the resulting acid-dependent electrochemistry can be found in later sections; however, a brief description of the data analysis is required. To determine more precisely the half wave potential of the redox processes of the vanadium oxide assembly, we invoked an approach reported by Vullev, in which the standard reduction potential is approximated by examining the second derivative of the cyclic voltammogram (CV).^39^ This method provides the added benefit of allowing for *E*_1/2_ values to be established for redox events whose irreversibility renders determination of half-wave potentials challenging. Following this procedure, we were able to construct the potential–p*K*_a_ diagram for **V_6_O_13_^–2^** as represented in Figure 1.

The electrochemical profile of **V_6_O_13_^–2^** in acetonitrile consists of two one-electron reduction events (*E*_1/2_ = −0.60 and −1.39 V vs Fc^+/0^).^40^ Addition of the weakest acids used in this study (organic acids with p*K*_a_ values >33) result in CVs that closely resemble that of **V_6_O_13_^–2^** collected in aprotic environments. This suggests that the reduced species is insufficiently basic to deprotonate organic acids with p*K*_a_ values higher than 33. This observation is correlated to the region in Figure 1 where no change in potential is observed (indicated by parallel horizontal lines).

Upon addition of organic acids with p*K*_a_ values between 19 and 33, significant changes to the redox properties of the cluster are observed. Across the range of acid strengths examined, the first reduction event remains reversible, with an *E*_1/2_ value independent of acid strength. Conversely, a loss of reversibility is observed in the second reduction event, suggesting that the increased basicity of the two-electron reduced cluster is sufficient to deprotonate the organic acid. This electrochemical behavior is reminiscent to that of quinone/hydroquinone systems measured in organic solvent;^41,42^ upon introduction of organic acids, a loss of reversibility is observed at the most reducing event as a result of the interaction of the acidic proton with the two-electron reduced form of the quinone.

**Scheme 1:**
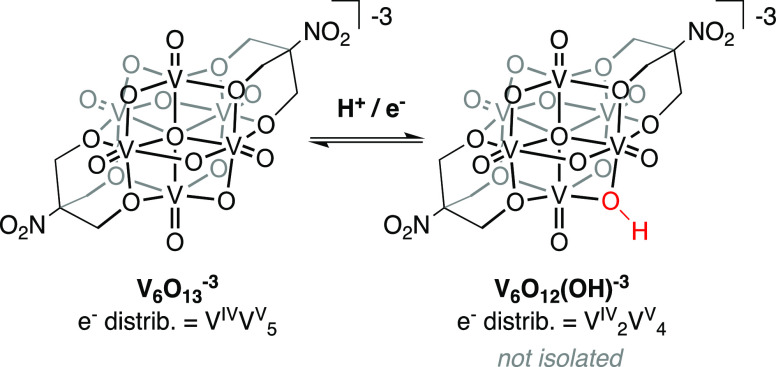
Proposed Reaction Scheme for the Formation of the Two-Electron Reduced, Singly Protonated Species Formed as a Result of the Reduction of **V_6_O_13_^–3^** in the Presence of an Acid with a p*K*_a_ Value in the Range of 19–30 in Acetonitrile This reaction is represented in Figure 1 as the sloped region between p*K*_a_ values of approximately 19 and 30.

Proton involvement in the charge transfer event is also suggested by the anodic shift of the reduction potential of the most-reducing redox event of **V_6_O_13_^–2^** as a function of the p*K*_a_ of the organic acid. Through use of the Nernst equation (eq 1; where *E*°’ is the predicted reduction potential, *E*° is the standard reduction potential of the cluster, *m* is the number of protons transferred, *n* is the number of electrons transferred, *R* is the universal gas constant, *T* is the temperature, *F* is Faraday’s constant, p*K*_a_(V_6_O_13–x_(*OH*)_*x*_)^*y*^ is the acid dissociation constant for a protonated version of the cluster, and p*K*_a_(HA) is the acid dissociation constant of the organic acid in acetonitrile), we are able to determine the stoichiometry of PCET at the surface of the cluster. A 1:1 ratio of protons to electrons being transferred will result in a shift of approximately 59 mV/p*K*_a_ unit. Fitting a linear trend, the sloped region of the experimental results by linear regression reveals a change in the reduction potential of ∼60 mV/p*K*_a_ unit. As such, we assign the second reduction process in the p*K*_a_ range of 19–33 as a one-electron, one-proton transfer event (Scheme 1). Despite electrochemical evidence for the formation of this reduced cluster, synthetic attempts to isolate and characterize this species were unsuccessful.
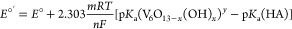
1

At p*K*_a_ values lower than 19, we observe a combination of the individual one-electron reduction processes into a single event. The difference in the maximum cathodic and anodic peak currents (Δ*E*_p_) varies across this range of acid strengths, where the observed difference is largest in the presence of the weakest acids (195 mV) and smallest with the strongest acids (162 mV). In all cases, Δ*E*_p_ is substantially larger than values predicted by the Nernst equation for two electron processes.^43^ Deviations from the expected Δ*E*_p_ values are commonly observed as a result of solution resistance; however, reversible multielectron transfer events typically retain separation values in the range of 30–70 mV.^44^ Differences much larger than these can be explained by an irreversible process, such as a chemical reaction accompanying reduction. It has been shown that quinones display multielectron/multiproton transfer events possess peak separations that are in the range of the values reported here (162–195 mV),^45^ suggesting the relatively large separation might be a result of a sluggish proton transfer event coupled to the electron transfer. Further evidence for this event being a multielectron process is obtained by comparing the redox events using square wave voltammetry (Figure S23). The relative integrations of isolated **V_6_O_13_^–2^** compared to the cluster in the presence of two equivalents of pyrazolium tetrafluoroborate (p*K*_a_ = 9.1) indicates that twice as much charge is passed in the acidified sample, providing support for multielectron transfer under these conditions.

Comparing the potential of the “two-electron” reduction event of **V_6_O_13_^–2^** in the presence of organic acids with p*K*_a_ values lower than 19, an anodic shift is observed. This change is similar to that observed for the acid dependent redox process of **V_6_O_13_^–2^** observed across the p*K*_a_ range of 33 to 19 (*vide supra*, ∼60 mV/p*K*_a_ unit). As previously mentioned, a slope of 59.1 mV/dec would be expected at room temperature for a 1:1 ratio of protons and electrons being transferred. Given the assignment of the redox process as corresponding to the transfer of two electrons, we pose that the electrochemical event observed for **V_6_O_13_^–2^** in the presence of organic acids with p*K*_a_ values less than 19 corresponds to a 2e^–^/2H^+^ coupled process.

Approximating the p*K*_a_ values for **V_6_O_11_(OH)_2_^–2^** and [V_6_O_12_(OH)(TRIOL^NO2^)_2_]^3–^ is possible using Figure 1**,** as the points where acid independent redox events (horizontal lines) intersect acid dependent redox events (diagonal lines). These “corners” indicate the point at which the basicity of the cluster is sufficiently strong, resulting in deprotonation of the acid in solution. These points have been marked by dashed lines in Figure 1 and represent the dissociation reactions shown in eqs 2 and 3 for p*K*_a1_ and p*K*_a2_, respectively.
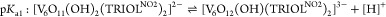
2
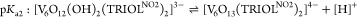
3

The results from the electrochemical experiments reveal acid dissociation constants of 19.3 and 32.7 for p*K*_a1_ and p*K*_a2_, respectively. These values indicate **V_6_O_13_^–2^** is quite basic in its reduced forms; particularly in comparison to the acid dissociation constants to values reported previously by our group for a series of similar Lindqvist-type POV-alkoxide clusters, [V_6_O_6_(OH)(OCH_3_)_12_]^*n*^ (*n* = −1, 0, +1; p*K*_a_ = 5.5–19.3).^14^ The differences in acid–base characteristics can be explained by the fact that protonation at this previously studied cluster is located at a terminal V=O site. Theoretical studies have shown that the relative basicity of oxide ligands in POMs is directly related to the number of metal centers bound to the oxygen atom, where the p*K*_a_ of the protonated species is found to increase as the number of metal-to-oxygen bonds increases.^46,47^

While *E*° and p*K*_a_ values describe the energy required for each step wise reaction, systems in which PCET is operative often utilize the energy required to form or break the E–H bond involved in the reaction. Typically, these parameters are reported as bond dissociation free energies (BDFEs). Due to the fact that both reduction and p*K*_a_ are free energy parameters, converting these to terms of BDFE(O-H) is accomplished by using the Bordwell equation (eq 4):
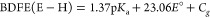
4where p*K*_a_ and *E*° are the acid dissociation and reduction potentials collected experimentally and *C*_g_ is a constant associated reduction of H^+^/H^●^ in the solvent used for the reaction (for acetonitrile, *C*_g_ = 52.6 kcal mol^–1^).^30^

**Scheme 2:**
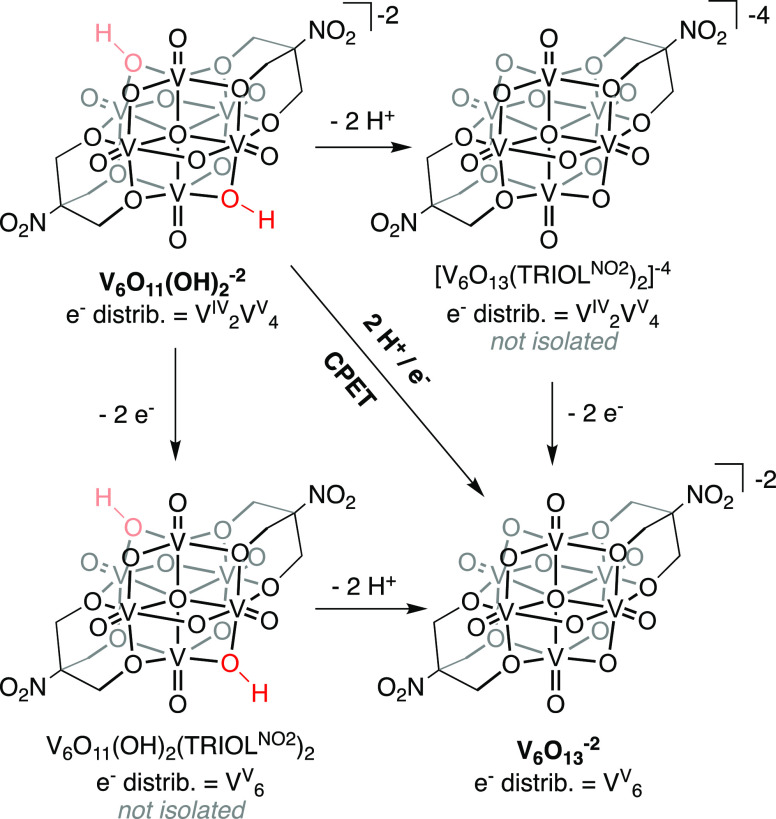
Square Scheme for the 2e^–^/2H^+^ Oxidation of **V_6_O_11_(OH)_2_**^**–2**^ The intermediate species containing odd numbers of protons or electrons have been omitted for clarity. The diagonal line connecting **V_6_O_11_(OH)_2_^–2^** and **V_6_O_13_^–2^** represents the direct formation of the oxidized product through the transfer of two H atom equivalents.

Using the aforementioned parameters, a BDFE(O-H)_avg_ of 65.3 kcal mol^–1^ is calculated for **V_6_O_11_(OH)_2_^–2^**. It is important to note that we are only able to report the average of the two O–H bonds broken upon oxidation of **V_6_O_11_(OH)_2_^–2^** to **V_6_O_13_^–2^** (Scheme 2). This is due to the fact that we are unable to observe and/or isolate monoprotonated species that would allow for the elucidation of individual BDFE(O-H) (see Scheme S1 for more information). Nonetheless, the average BDFE(O-H) value allows for predictions to be made of relevance to the driving force of PCET reactions at **V_6_O_13_^–2^**, due to the fact that the reactivity of reagents that donate multiple H atom equivalents are dictated by the average of the E-H bonds broken or formed in the reaction.

In an effort to experimentally confirm the stoichiometry and BDFE(O-H)_avg_ of PCET from **V_6_O_11_(OH)_2_^–2^** under these reaction conditions, we turned to methods recently reported by Mayer and coworkers.^30^ Through the use of open circuit potential (OCP) analysis, the number of H atom equivalents can be extracted, through the use of eq 5:
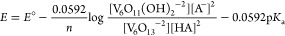
5where *E* is the predicted potential, *E*° is the standard reduction potential, *n* is the number of H atom equivalents, [**V_6_O_11_(OH)_2_^–2^**] and [**V_6_O_13_^–2^**] are the concentration of the reduced and oxidized cluster, respectively, [A^–^] and [HA] are the concentration on the conjugate acid–base pair used as buffer in solution, and p*K*_a_ is the acid dissociation constant of the acid used in the buffer. Notable in this derivation of the Nernst equation, is the fact that *both* protons and electrons will impact the slope of the curve, allowing for differentiation between equal ratios of protons and electrons being transferred.

**Figure 2:**
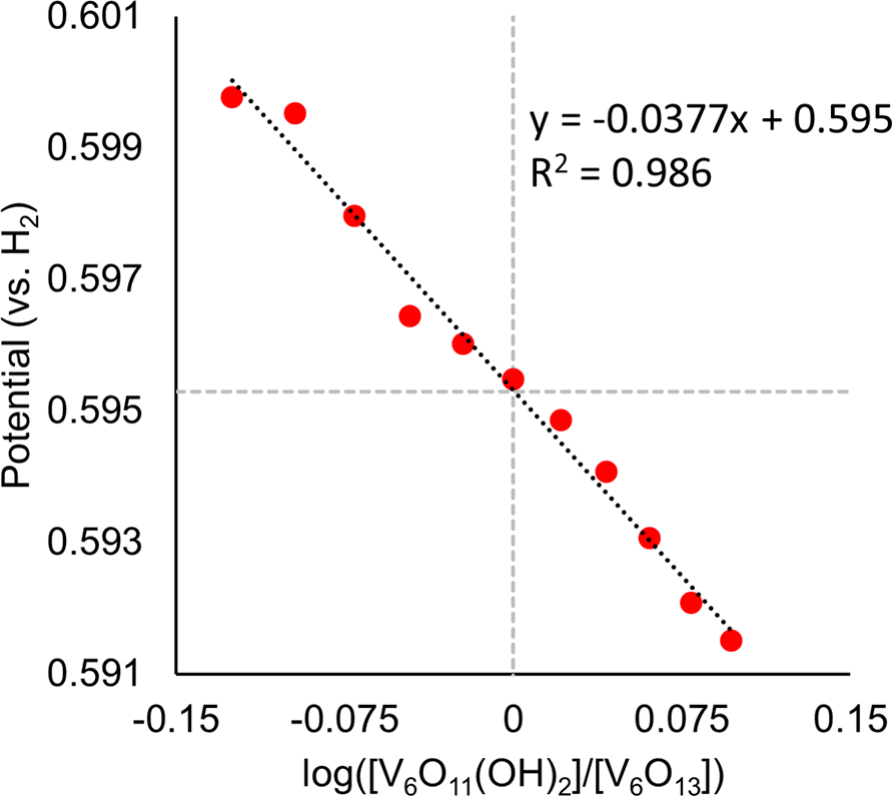
Plot of the open circuit potentials measured at various ratios of **V_6_O_11_(OH)_2_^–2^/V_6_O_13_^–2^**, against the log of the ratio of the clusters referenced against H_2_. All measurements were performed in acetonitrile containing a 0.05 M buffer of 1:1 DMAH^+^/DMA (p*K*_a_(DMAH^+^) = 11.5^9^) and supporting electrolyte (0.1 M [^n^Bu_4_N][PF_6_]). The slope of the linear trend closely resembles the value expected by the Nernst equation (eq 2) for a two-electron, two-proton transfer event. From the *y*-intercept (marked with dotted gray lines), the BDFE(O-H)_avg_ for **V_6_O_11_(OH)_2_** can be calculated using eq 6, with *E*°(X/XH_*n*_) as the y-intercept (0.595 V).

OCP analyses were run in the presence of an excess of buffer with a p*K*_a_ value between 9 and 19, as this p*K*_a_ corresponds to the the region of the diagram that supports the transfer of both H atoms between the cluster (for more information, see Experimental Section). Plotting the resultant data as the potentials measured against the log of the ratio of reduced to oxidized cluster present in solution (Figure 2), a trend line was found using least square fitting on the best fit line, resulting in a slope of −0.037 ± 3.3 V/dec. While this value deviates from that predicted by the Nernst Equation (0.0296 V/dec), it is comparable to values reported by both our group^27^ and the Mayer group,^30^ in which multielectron-multiproton reactivity was established.

Measuring the change in the open circuit potential of a sample while varying the ratio of oxidized to reduced cluster allows for the direct measurement of the free energy required for the transfer of two H atom equivalents at the surface of **V_6_O_13_^–2^**. By referencing the potentials measured against the H^+^/H_2_ couple, the average bond strength of the two hydroxide ligands can be found using eq 6, where *E*°(X/XH) is equal to the open circuit potential measured for a 1:1 mixture of reduced and oxidized cluster (the *y-*intercept in Figure 2), and Δ*G*° is a constant related to the free energy required to homolytically cleave H_2_ in acetonitrile (ΔG° = 52 kcal/mol^30^). Using this method, we find a BDFE(O-H)_avg_ of 65.7 ± 0.005 kcal mol^–1^, in good agreement with the value obtained from the potential–p*K*_a_ diagram (65.3 kcal/mol), suggesting the potential–p*K*_a_ method is a viable method to accurately determine the O–H bond strengths at POM surfaces.
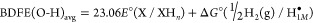
6

Despite the fact that acid-dependent redox chemistry of polyoxometalates has been studied since their discovery, there has been limited effort made toward the quantification of O–H bond strength at the surface of reduced assemblies. In fact, to the best of our knowledge, there are only two examples. The first report analyzed the thermochemistry of C–H oxidation reactions of a divanadium-doped polyoxotungstate cluster; HAT to the metal oxide surface resulted in the formation of a single bridging hydroxide ligand between the two vanadium centers.^26^ The authors determined the BDFE(O-H) as 72.6 kcal mol^–1^, significantly larger than the BDFE(O-H)_avg_ measured for **V_6_O_11_(OH)_2_^–2^**. While it has been shown in extended state metal oxide systems that differences in BDFE values arise as a result of defect sites at the surface of the material,^48^ the fact that in both systems, atomically precise polyoxometalates are used suggests that this difference originates from a different reason. We justify the difference in BDFE(O-H) of these systems as a result of the extent of charge distribution across the metal oxide core; in the case of [PV_2_W_10_O_40_]^−6^, the electron is localized between the two vanadium centers. Localization of the electron to a site tangential to that of the proton allows for a more substantial coupling effect, resulting in a larger BDFE(O-H). Indeed, reduction of **V_6_O_13_^–2^** results in an electron that is delocalized across the Lindqvist ion.^49^

The second example of the quantification of bridging O–H bond strengths at reduced polyoxometalates comes from previous work from our group, in which BDFE(O-H)_avg_ corresponding to cleavage of two O–H moieties of the six-electron, six-proton reduced cluster [V_6_O_7_(OH)_6_(TRIOL^NO2^)_2_]^−2^ was determined. Similar open circuit potential methods were used in order to establish the BDFE(O-H)_avg_ corresponding to removal of the first two H atoms as 61.6 ± 0.009 kcal mol^–1^.^27^ Comparing this value to the BDFE(O-H)_avg_ determined here for **V_6_O_11_(OH)_2_^–2^** allows us to establish the connection between the oxidation state distribution of vanadium centers within the cluster core and the strength of O–H bonds at the surface of the assembly. The more oxidized cluster, **V_6_O_11_(OH)_2_^–2^** (e^–^ distrib: V^IV^_2_V^V^_4_), forms more thermodynamically stable hydroxide ligands compared to the more reduced vanadium oxide assembly, [V_6_O_7_(OH)_6_(TRIOL^NO2^)_2_]^−2^ (e^–^ distrib: V^IV^_6_). This result is similar to those observed in nanocrystalline metal oxide systems, in which the BDFE(O-H) of surface bound H atoms are directly dependent upon the extent of reduction at the metal centers of the nanoparticle.^50^

**Figure 3:**
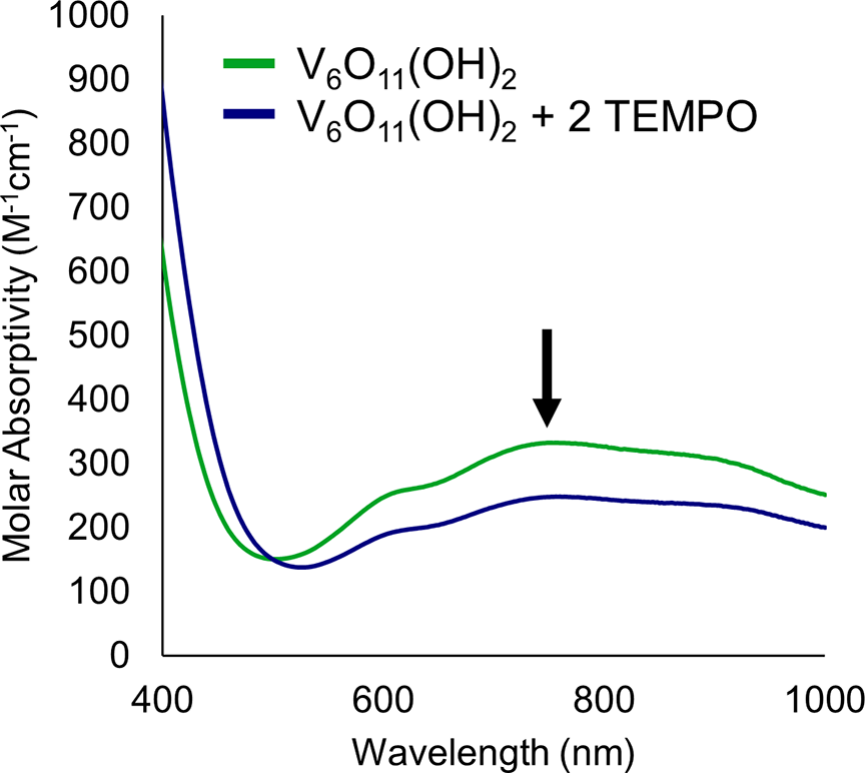
Comparison of electronic absorption spectra for the isolated reduced cluster, **V_6_O_11_(OH)_2_^–2^** (green), and the resulting spectra for the mixture of **V_6_O_11_(OH)_2_^–2^** and two equivalents of TEMPO (blue). A sample containing 0.75 mM **V_6_O_11_(OH)_2_^–2^** was prepared in acetonitrile, to which two equivalents of TEMPO was added and allowed to reach equilibrium at 25 °C over 30 min.

The average BDFE(O-H) value of the surface hydroxide ligands of **V_6_O_11_(OH)_2_^–2^** indicates relative lability of H atoms in comparison to a comprehensive library of PCET reagents.^8,51^ Accordingly, we evaluated the ability of **V_6_O_11_(OH)_2_^–2^** to donate H atoms to a substrate. Upon introduction of two equivalents of TEMPO (2,2,6,6-tetramethylpiperidin-1-yl)oxyl; BDFE(O-H) = 66 kcal mol^–1^^8^) to **V_6_O_11_(OH)_2_^–2^**, the color of the solution changes from green to yellow. Determination of the extent of reactivity is possible through comparison of electronic absorption spectra; the relative concentration of reduced to oxidized cluster in solution was obtained by assessing the change in molar absorptivity of the IVCT band characteristic of the reduced cluster, **V_6_O_11_(OH)_2_^–2^** (upon oxidation to **V_6_O_13_^–2^** this peak is no longer observed; Figure 3 and Figure S24). From this data, we are able to calculate the BDFE(O-H)_avg_ as 66.1 kcal mol^–1^, in good agreement with the values determined electrochemically (see the SI).

### Predicting Rates of PCET from the POV-Alkoxide Surface Using Marcus Theory

The traditional method for deriving the relationship between the overall driving force and the rate of charge transfer reactions is through the use of Marcus theory.^52−54^ This theory has been demonstrated applicable in nearly every area of chemistry (e.g., photosynthesis, corrosion, chemiluminescence, charge separation, heterogeneous charge transfer), providing insight into self-exchange rate constants, the relation between driving force and rate constants, impact of solvent on reaction, and the relationship between self-exchange constants and rate of charge transfer between and electrode and substrate. Although originally designed to describe the rates of reactions for outer sphere electron transfer, this theory has been reported to successfully predict the rate constants for S_N_2 reactions and proton transfer.^55,56^ Of interest to our work is the emergence of using Marcus theory to describe the kinetics of PCET over the last 20 years; a series of reports by Mayer and coworkers demonstrated that Marcus theory, and in particular the Marcus cross relation, was able to accurately predict the rate constant for PCET reactions for a variety of organic substrates and monometallic transition metal complexes capable of performing one electron one-proton transfer within 1 or 2 orders of magnitude.^33,57^ The ability to correlate the driving force to rate constants allows for a bridge between theory and experiment, leading to the design of systems capable of the efficient transfer of H atoms. Additionally, the use of Marcus–Hush theory enables researchers to obtain further kinetic parameters (e.g., reorganization energy) that dictate the transfer of hydrogen atom equivalents between donor and acceptor molecules.

We were inspired to apply this theory to the clusters studied here in order to establish the ability for Marcus theory to predict the kinetics of PCET at POM surfaces. Similar to Mayer,^33^ our efforts to determine the ability to describe kinetics at POMs using Marcus theory revolve around the use of the Marcus cross relation, shown in eq 7. In this equation, the rate constant for PCET at the surface of the clusters, *k*_calc_, can be determined from the H atom self-exchange constants, *k*_XH/X_ and *k*_YH/Y_, the equilibrium constant *K*_eq_, and the factor, *f*, which is defined in eq 8, where *Z* is the effective collisions of particles in solution (typically taken as 10^11^ M^–1^ s^–1^). The equilibrium constant can be determined through the overall change in free energy of the reaction through eq 9.
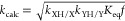
7
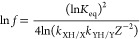
8
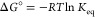
9

It is important to note, that in systems that Marcus theory is typically used to describe the kinetics of the transfer of one electron and one proton. In **V_6_O_11_(OH)_2_^–2^**, there are two H atom equivalents being transferred. Ideally, the *K*_eq_ will reflect this by using the BDFE(O-H) of the hydroxide ligand broken/formed at the rate-limiting step of the reaction. However, despite extensive efforts, we were unable to isolate the 1e^–^/1H^+^ reduced cluster. As a result, we are unable to calculate the precise *K*_eq_ for the reaction and have elected to use the overall free energy change of the 2e^–^/2H^+^ transfer. While this fact likely introduces error into our calculations, this bias is consistent across all reactions reported.

In order to calculate rate constants for a series of PCET reactions, we use the modified version of the cross relation, eq 10. From this, we are able to predict the value of the rate constant for PCET based on the relative difference in the driving force when compared to a reaction with a known rate constant. In this study, we use the reaction between **V_6_O_11_(OH)_2_^–2^** and TEMPO as the anchor point, with which we predict every other rate constant. This equation can be further simplified by eliminating like terms and assuming the factor *f* is near unity, resulting in eq 11, where *k*_calc_ is the calculated rate constant of the reaction of interest, *k*_TEMPO_ is the rate constant for the reaction between **V_6_O_11_(OH)_2_^–2^** and TEMPO, *k*_YH/Y_ is the self-exchange constant for the substrate of interest, *K*_eq_ is the equilibrium constant of the reaction of interest, *k*_TEMPO/H_ is the self-exchange constant of TEMPO, and *K*_TEMPO_ is the equilibrium constant between **V_6_O_11_(OH)_2_^–2^** and TEMPO.
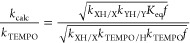
10
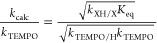
11

**Figure 4:**
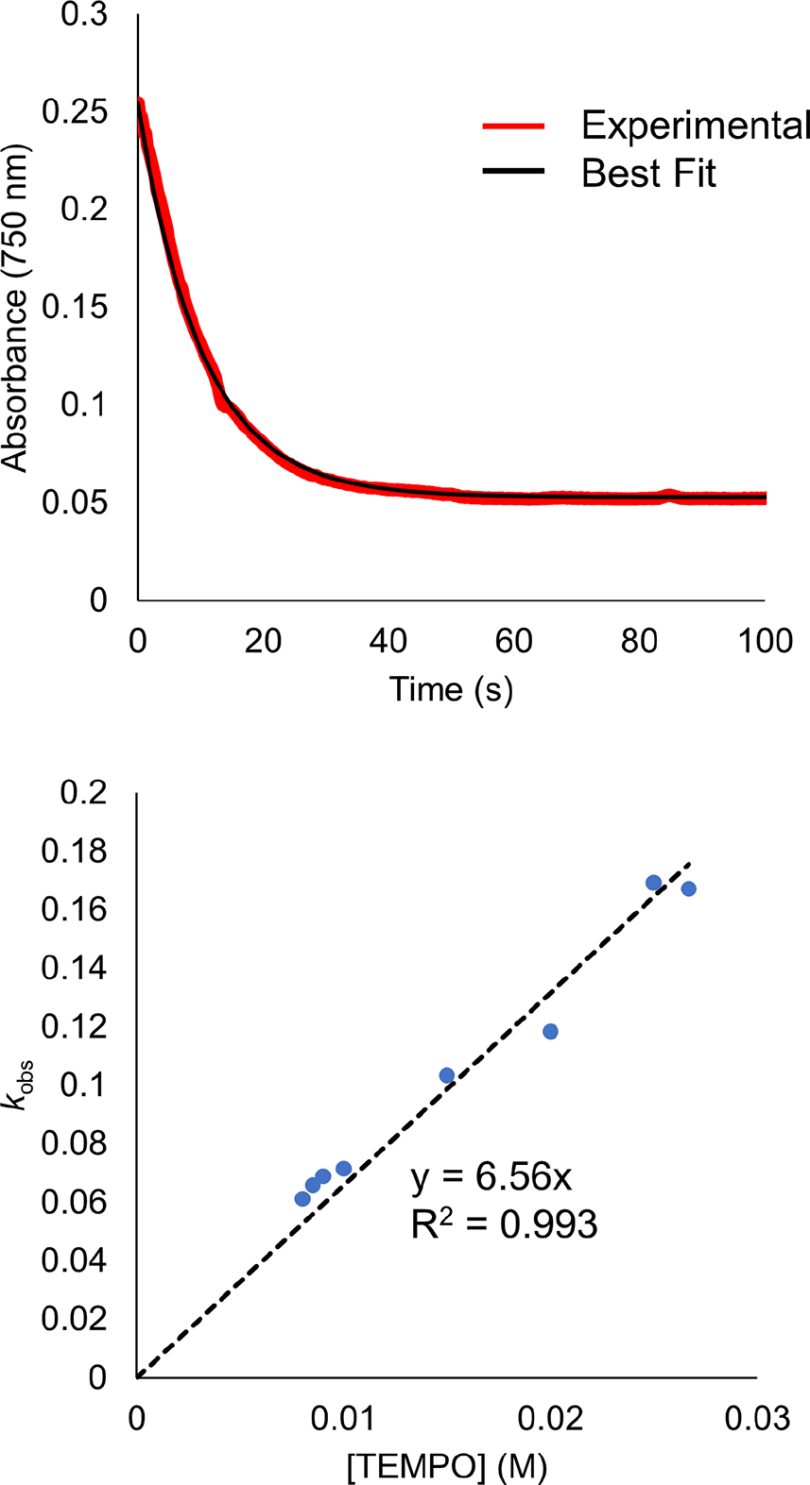
(Top) Kinetic trace following the reaction between **V_6_O_11_(OH)_2_^–2^** and TEMPO. The reaction is run in pseudo-first-order reaction conditions in acetonitrile at 25 °C, with 0.5 mM **V_6_O_11_(OH)_2_^–2^** and 15 mM TEMPO; (bottom) plot of the observed rate constants for the reaction between **V_6_O_11_(OH)_2_^–2^** and TEMPO against the concentration of TEMPO initially in solution. The black dotted line represents the best fit line with a *Y* intercept of 0. *k*_exp_ can be found from the slope of the graph, where *k*_exp_ = ^1^/_2_ × slope, where the ^1^/_2_ accounts for the two equivalent bridging hydroxide ligands at the surface of the cluster. Each reaction is run at 25 °C in acetonitrile under pseudo-first-order reaction conditions with 0.5 mM **V_6_O_11_(OH)_2_^–2^**.

Electronic absorbance spectroscopy was used to measure the extent of oxidation of the cluster by TEMPO over time. Kinetic experiments were performed under pseudo-first-order reaction conditions, with the concentration of TEMPO in at least 10-fold excess relative to the cluster in solution. Monitoring the absorbance of the reaction mixture at 750 nm over time yields a trace such as that found in Figure 4 (top). From this data, the observed rate constant in pseudo-first-order reaction conditions can be obtained by finding the best fit of a trace through least-squares fitting (see the SI).

To obtain the rate constant for the reaction between the cluster and TEMPO, the pseudo-first-order kinetic reactions were repeated using a range of concentrations of TEMPO (10–30 mM). Plotting the resultant observed rate constants against the concentration of TEMPO reveals a linear trend (Figure 4, bottom), indicating that the order with respect to TEMPO in this reaction is 1. The overall second order rate constant for the reaction between **V_6_O_11_(OH)_2_^–2^** and TEMPO can be found as slope = 2*k*_exp_, where the coefficient 2 accounts for the two identical O-H ligands found at the surface of **V_6_O_11_(OH)_2_^–2^**. From the data collected here, the rate constant for PCET from is determined (*k*_exp_ = 3.3 ± 0.2 M^–1^ s^–1^).

With the experimental rate constant for the reaction between **V_6_O_11_(OH)_2_^–2^** and TEMPO determined, we are able to predict rate constants for a series of reactions using eq 11. While there is no shortage of organic PCET substrates with reported BDFE values in organic solvents, we are limited to reagents with reported rate constants of H atom self-exchange.^58,59^ With this constraint in mind, we narrowed our investigation to the reagents listed in Table 1; values for the reduced versions of the substrates span 9 kcal mol^–1^, providing opportunities for the analysis of the rate of PCET over a range of driving forces.

**Table 1:** Thermochemical and Kinetic Parameters Used to Calculate the Rate Constant between either **V_6_O_13_^–2^** and **V_6_O_11_(OH)_2_^–2^** and HAT Reagent

substrate	BDFE (kcal mol^–1^)^a^	*k*_YH/Y_ (M^–1^ s^–1^)^c^	*k*_calc_ (M^–1^ s^–1^)	*k_exp_* (M^–1^ s^–1^)	*k*_relative_^e^
TEMPO^•^	66	4.7	3.5	3.3	1.06
iAscH^–^	67.3	5 × 10^5^	109.9	115.7	1.05
H_2_Q	74.2^b^	1719^d^	1.8 × 10^–2^	4.4 × 10^–3^	4.1
anthracene	75	5 × 10^–11^	2.4 × 10^–3^	4.0 × 10^–4^	6.0

a Unless noted, all values are reported in acetonitrile, from ref (33).

b BDFE converted from DMSO using methods from ref (33).

c Unless noted, all self-exchange values are reported in acetonitrile, from ref (58).

d Value reported in CCl_4_ from ref (57), converted to MeCN using methods from ref (58).

e *k*_relative_ is determined by *k*_calc_/*k*_exp_ or *k*_exp_/*k*_calc_, whichever method produces a value greater than 1, in line with examples published previously by the Mayer group.^58^

To confirm these predicted values, we measured the rate of reduction of **V_6_O_13_^–2^** by 1,4-hydroquinone (H_2_Q). In a similar manner to the experiments focused on the oxidation of **V_6_O_11_(OH)_2_^–2^** by TEMPO, the rate of reaction between **V_6_O_13_^–2^** and H_2_Q is measured via electronic absorption spectroscopy. Upon introduction of the oxidized cluster to 50 equivalents of H_2_Q in acetonitrile, the formation of the reduced assembly occurs over approximately 1 h, significantly slower than the experiments focused on PCET from **V_6_O_11_(OH)_2_^–2^** to TEMPO. The sluggish kinetics of this reaction is expected as even though the average BDFE(O-H) of H_2_Q is reported as only 2 kcal mol^–1^ larger than the average for **V_6_O_11_(OH)_2_^–2^**, the free energy of the first O–H bond broken at H_2_Q in this reaction is reported as 74.2 kcal mol^–1^ in acetonitrile, indicating an energetically uphill process as the rate-determining step of this reaction.

We next measured the rate of reaction between **V_6_O_13_^–2^** by the reductant tetrabutylammonium 5,6-isopropylidine ascorbate (iAscH^–^). From Table 1, the calculated rate constant suggests that this reaction occurs rapidly in comparison to the reaction of the reduced assembly, **V_6_O_11_(OH)_2_^–2^**, with either TEMPO or H_2_Q. As such, obtaining an experimental rate constant for the reaction between iAscH^–^ and **V_6_O_11_(OH)_2_^–2^** necessitated decreasing the temperature of the reaction. Repeating the kinetic experiments at −40 °C results in a change in absorbance over time that can be clearly resolved, allowing for the extraction of the observed rate constant by fitting a model to the experimental data. Eyring analysis allows for the extrapolation *k*_exp_ to 25 °C, where we have found the value to be 115.7 ± 0.2 M^–1^ s^–1^ (Figure S26). When comparing the calculated and measured rate constants, a relative value of 1.05 is obtained.

The final reaction evaluated is the reduction of anthracene by **V_6_O_11_(OH)_2_^–2^**. While this compound has a significantly larger BDFE(E-H) value compared to that of the cluster, PCET reagents containing H atoms bound to carbon typically experience substantially small self-exchange constants as a result of the non-polar C–H bond. This lack of polarity prevents the organization of H atom donor and acceptor for HAT.^31,32,60^ This sluggish behavior is reflected in the small calculated rate constant, despite the fact that dihydroanthracene possess a BDFE(C-H) value that is ∼10 kcal mol^–1^ larger than the BDFE(O-H)_avg_ of the reduced cluster.

Initial attempts to collect the rate constant for this reaction revealed inconsistent results, where the plot of the observed rate constant in pseudo-first-order reaction conditions against the concentration of anthracene in acetonitrile did not possess a discernable trend (Figure S27). The most likely reason for this observation is O_2_ contamination of the sample over the long reaction period. In order to prevent this side reaction, we monitored the reaction instead via ^1^H NMR spectroscopy, where the samples are sealed in air-tight J-Young tubes. The concentration of cluster as a function of time was determined by comparing the integration of a signal belonging to **V_6_O_13_^–2^** to that of an internal standard (hexamethyldisiloxane; see the SI). When the resulting *k*_obs_ value is plotted against the concentration of anthracene present at the beginning of the reaction, the linear trend with a *Y* intercept of 0 has a poor fit to the experimental data (Figure S28). We instead plotted as the natural log of *k*_obs_ against the natural log of the concentration of the initial concentration of anthracene, revealing a linear trend with a slope of approximately 2. This suggests the order with respect to anthracene is in fact 2 (Figure S29). From the *Y* intercept, we are able to obtain the *k*_exp_ value as 4.0 (± 2.7) × 10^–4^ M^–2^ s^–1^.

In comparing this value to that predicted using eq 11, we find a relative value of 6.0. Despite apparently deviating in mechanism, the experimental rate constant is in good agreement with the value predicted by the modified version of the Marcus cross relation, suggesting that the driving force of the rate-limiting step of this reaction is dependent upon the difference in BDFE(E-H) between the cluster and substrate. This observation is consistent with a mechanism that consists of a concerted transfer of protons and electrons to anthracene, as a mechanism in which the proton and electron are transferred in separate steps will result in driving forces that are dependent upon the relative p*K*_a_ values or reduction potentials.^61^

**Figure 5:**
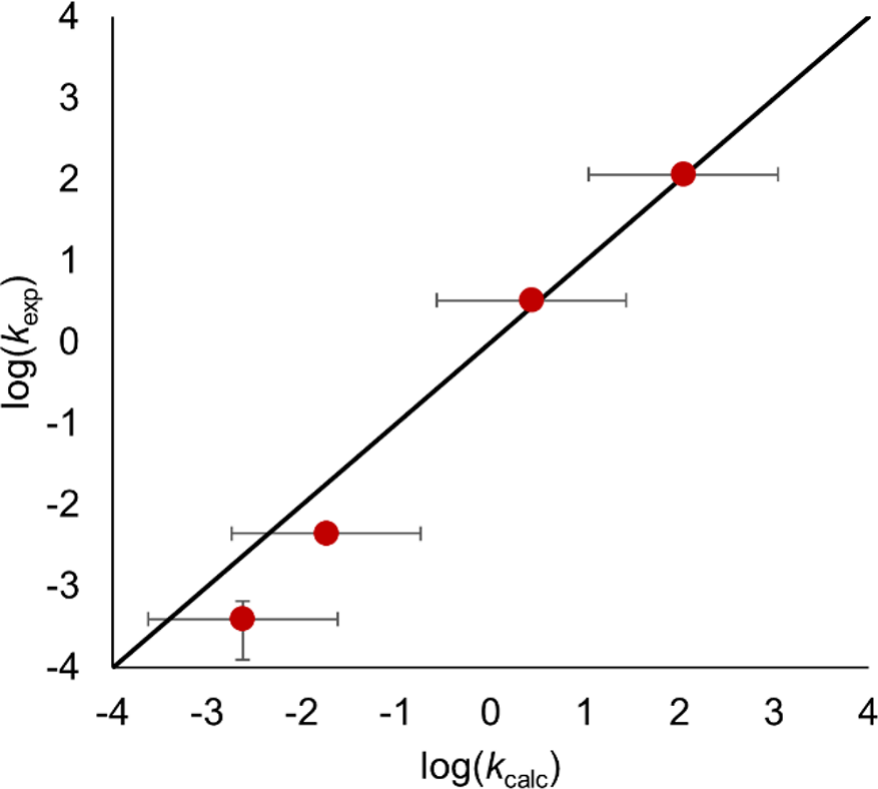
Plot of the correlation between the measured rate constant for PCET at the POV cluster, *k*_exp_, plotted against the value predicted by eq 11, *k*_calc_. The solid black line indicates unity between the measured and predicted values. The horizontal error bars represent one order of magnitude, which has been used previously as the range with which this method is accurate toward predicting values of rate constants for PCET. Vertical error bars represent the error reported in this work.

The agreement between the values predicted using the modified version of the Marcus cross relation and the experimentally observed values can be seen in Figure 5, where the solid black line represents perfect agreement between computed and experimentally obtained values. Each predicted value is within an order of magnitude of the value measured experimentally, with an average deviation of 3.05. The substrates used in this study span several orders of magnitude in both hydrogen atom self-exchange rates and BDFE(E-H) of the reduced compound. This suggests that this method is a robust method for deriving the rate constants for PCET at molecular metal oxide surfaces. Notably, the stoichiometry of PCET is shown to not negatively impact the ability to study the rate of reaction, as our results show reliability for both single and multi-HAT reagents. These results are significant for metal oxide surfaces, where multiproton/multielectron transfer chemistry is inherently operative. It is important to note, however, that the cross relation is a simplified conceptual model, that neglects factors such as electronic and vibronic coupling that have been shown by the Hammes–Schiffer group to be critical in fully understanding the kinetics of PCET.^62,63^ While the cross relation has been shown in this work, and previous studies, to be effective in predicting the rate constant of PCET to within an order of magnitude, it is not sufficient for fully describing certain aspects of PCET, such as kinetic isotope effects. As such, it is important to use caution when relying on this theoretical method to describe the kinetics of charge transfer at metal oxide compounds.

## Conclusions

We have demonstrated multiple methods for elucidating the thermochemical parameters involved in the concerted transfer of protons and electrons at the surface of POMs. The first method described involves measuring the electrochemical response of the cluster while in the presence of a series of organic acids. As the strength the acid is increased, the energy required for reduction is decreased as a result of concomitant protonation occurring at the surface of the cluster. Most notable is the fact that upon plotting the redox potential against the p*K*_a_ of the acid, each individual thermochemical parameter for PCET at the cluster can be determined without the need for isolation of each intermediate involved in the reaction. From these parameters, the BDFE(O-H)_avg_ for **V_6_O_11_(OH)_2_^–2^** was calculated as 65.3 kcal mol^–1^. In a subsequent set of electrochemical analyses, a series of open circuit potential measurements were collected of samples containing a range of mixtures of reduced and oxidized versions of the cluster. This direct measurement of BDFE(O-H)_avg_ is in good agreement with the previous value (65.7 kcal mol^–1^). Additionally, the BDFE(O-H)_avg_ of **V_6_O_11_(OH)_2_^–2^** can be determined directly from the equilibrium of the cluster in the presence of a PCET reagent, where the relative concentration of oxidized and reduced cluster allows for the equilibrium constant to be obtained. In all three experiments, the resulting BDFE(O-H) values agree with each other, suggesting either is appropriate for determining the free energy required for PCET to occur at polyoxometalate clusters.

The ability to thermochemically describe the transfer of hydrogen atom equivalents at metal oxide surfaces is valuable, as despite the fact that PCET has been shown to be active at polyoxometalate clusters for decades, very few studies have reported these values. However, it is important to note that the driving force of the reaction is often insufficient to fully describe the ability for HAT to occur. For example, in homogeneous, molecular PCET reactions, factors such as intrinsic barriers and activation energies play a significant role in the ability for these charge transfer reactions to occur. Notably, both of these reaction parameters are unable to be determined through changes in free energy alone. Although Marcus theory has primarily been used to correlate driving force to kinetics for electron transfer, recent efforts have demonstrated the applicability of this theory to systems in which both electrons and protons are transferred. In this report, we establish a direct relationship between the overall change in free energy for HAT at the surface of a metal oxide assembly to the rate of the said reaction. Using a modified version of the Marcus cross relation, we are able to accurately predict the rate constants for a series of multiproton/multielectron PCET reactions at a POV cluster. This work is important as the design of increasingly efficient systems in which metal oxide compounds are required to transfer both protons and electrons relies on the ability to describe both the thermodynamics and kinetics of HAT. Additionally, Marcus theory enables researchers to establish parameters that are often challenging to obtain, such as the intrinsic barrier, allowing for a more in-depth understanding as to how PCET occurs at an atomic level in polyoxometalates, and more broadly, at the surface of extended metal oxides.

## Supporting Information Available

Experimental procedures, cyclic voltammograms for acid titration experiments, electronic absorption spectra for polyoxovanadate-alkoxide clusters, and additional kinetic results used to determine rate constants of hydrogen atom transfer (PDF)
