# Supplementary material for: Connecting Thermodynamics and Kinetics of Proton Coupled Electron Transfer at Polyoxovanadate Surfaces Using the Marcus Cross Relation
Source: Inorg Chem. 2022 Sep 1;62(5):1958–67. doi: 10.1021/acs.inorgchem.2c02541 (PMC9906739; doi:10.1021/acs.inorgchem.2c02541)
Supplement: Supplementary file 1 — ic2c02541_si_001.pdf [file ic2c02541_si_001.pdf]

Electronic Supporting Information

Connecting Thermodynamics and Kinetics of Proton Coupled Electron Transfer at Polyoxovanadate Surfaces using the Marcus Cross Relation

Alex A. Fertig, Ellen M. Matson*

*Corresponding Author E-mail: matson@chem.rochester.edu

Department of Chemistry, University of Rochester, Rochester NY, USA 14627

Supporting Information Table of Contents

Experimental S2
Table S1. Organic acid pK_a values S6
Figure S1-S22. CV of $\mathbf{V_6O_{13}}^{-2}$ in the presence a of organic acids S7-S12
Figure S23. Square wave voltammograms for $\mathbf{V_6O_{13}}^{-2}$ in the absence and presence of acid S13
Figure S24. Electronic absorption spectra for $\mathbf{V_6O_{13}}^{-2}$ and $\mathbf{V_6O_{11}(OH)_2}^{-2}$ S13
Figure S25. k_{obs} vs. [H_2Q] for reaction of $\mathbf{V_6O_{13}}^{-2}$ with H_2Q S14
Figure S26. Eyring plot for reaction between $\mathbf{V_6O_{13}}^{-2}$ and $iAscH^{-1}$ S14
Figure S27. Kinetic results of $\mathbf{V_6O_{11}(OH)_2}^{-2}$ and anthracene collected by UV-Vis S15
Figure S28. k_{obs} vs. [anthracene] for reaction of $\mathbf{V_6O_{11}(OH)_2}^{-2}$ with anthracene S15
Figure S29. Plot of ln(k_{obs}) vs. ln[Anthracene] for reaction of $\mathbf{V_6O_{11}(OH)_2}^{-2}$ with anthracene S16
References. S17

Experimental

General Considerations. All manipulations were carried out in the absence of water and oxygen using standard Schlenk techniques, or in a UniLab MBraun inert atmosphere drybox under a dinitrogen atmosphere. All glassware was oven dried for a minimum of 4 hours and cooled in an evacuated antechamber prior to use in the drybox. Solvents were dried and deoxygenated on a Glass Contour System (Pure Process Technology, LLC) and stored over 3 Å molecular sieves purchased from Fisher Scientific and activated prior to use. Hydrazobenzene and tetrafluoroboric acid diethyl ether complex were ordered from Sigma Aldrich and used as received. $\mathbf{V_6O_{13}^{-2}}$ was prepared according to previously reported procedures.[1]

^{1}H NMR spectra were recorded at 400 MHz or 500 MHz on a Bruker DPX-400 or Bruker DPX-500 spectrometer locked on the signal of deuterated solvents. All chemical shifts were reported relative to the peak of residual H signal in deuterated solvents. CD_3CN was purchased from Cambridge Isotope Laboratories, degassed by three freeze−pump−thaw cycles, and stored over fully activated 3Å molecular sieves. Electron absorption measurements were recorded in anhydrous acetonitrile in a sealed 1 cm quartz cuvette with an Agilent Cary 60 UV-Vis spectrophotometer. All kinetic experiments were held at the desired temperature using a Unisoku CoolSpek UV cryostat for UV-Vis spectrophotometry.

Cyclic voltammetry experiments were recorded with a Bio-Logic SP-150 potentiostat/galvanostat and the EC-Lab software suite. All measurements were performed in a three-electrode system cell configuration that consisted of a glassy-carbon (ø = 3.0 mm) as working electrode (CH Instruments, USA), a Pt wire as the counter electrode (CH Instruments, USA), and an Ag/Ag^+ non-aqueous reference electrode with 0.01 M $AgNO_3$ in 0.05 M [^{n}Bu$_4$N][PF$_6$] in acetonitrile (BASi, USA). The supporting electrolyte, [^{n}Bu$_4$N][PF$_6$] was purchased from Sigma-Aldrich, recrystallized three times using hot ethanol, and stored under dynamic vacuum for a minimum of two days prior to use. All electrochemical measurements were performed at room temperature in a nitrogen-filled drybox.

Synthetic methods.

*Synthesis of [^{n}Bu$_4$N]$_2$[$V_6O_{11}(OH)_2$(TRIOL^{NO2})$_2$] (**$V_6O_{11}(OH)_2^{-2}$**).* A 20 mL scintillation vial was charged with [^{n}Bu$_4$N]$_2$[V_6O_{13}(TRIOL^{NO2})$_2$] (0.056 g, 0.043 mmol) and 5 mL of DCM. Hydrazobenzene (0.008 g, 0.043 mmol) was added with stirring. A gradual color change from yellow to green was observed over the course of 5 minutes; the solution was left to stir for 30 minutes to ensure completion of the reaction. Volatiles were removed under reduced pressure, resulting in the isolation of a green solid. The product was washed with (x3) 10 mL diethyl ether to remove azobenzene, affording the isolation of the product, [^{n}Bu$_4$N][$V_6O_{11}(OH)_2$(TRIOL^{NO2})$_2$] in good yield (0.046 g, 0.035 mmol, 81%). UV-Vis [ε (M^{-1} nm^{-1}]: 772 (722). Elemental Analysis: Calcd for $V_6O_{23}C_{40}H_{88}N_4$ • 1.25 CH_2Cl_2 (MW = 1402.94 g mol^{-1}) C, 35.32; H, 6.36; N, 3.99. Found: C, 35.207; H, 6.220; N, 3.998.

General Procedure for Synthesis of Organic Acids: A 20 mL scintillation vial was charged with the conjugate base and 5 mL of diethyl ether (Et_2O). 0.95 equivalents of tetrafluoroboric acid diethyl ether complex was added over 5 minutes with intense stirring. Note: acidification of organic compounds is a highly exothermic process, as such care must be taken to not allow reaction to boil over. The solution is left to stir for 10 minutes, where a white precipitate is formed. The supernatant is removed and 10 mL of fresh Et_2O is added to the vial containing the white solid and stirred vigorously for 10 minutes to remove any unreacted conjugate base. The white solid is filtered off and washed with 10 mL Et_2O. Volatiles were

removed under reduced pressure over night, resulting in the isolation of a white solid in quantitative yield. Each acid is stored inside a nitrogen filled glovebox in a desiccator containing P_2O_5 in order to keep the sample anhydrous for long-term storage.

General Procedure for Performing Cyclic Voltammetry Experiments. A 5 mL sample was prepared in acetonitrile containing 0.5 mM $\mathbf{V_6O_{13}}^{-2}$ and 0.1 M [^{n}Bu$_4$N][PF$_6$] as a supporting electrolyte. An initial cyclic voltammogram was collected to ensure purity of the sample. An aliquot of a stock solution containing the desired organic acid was added to the sample and allowed to stir for 5 minutes, whereupon a cyclic voltammogram was collected. At completion of each experiment, a sample of ferrocene was added and an additional cyclic voltammogram was collected in order to properly reference to the $Fc^{+/0}$ couple.

General Procedure for Performing Open Circuit Potential Experiments. This procedure is adapted from previously reported methods.[2] A 5 mL sample was prepared in acetonitrile containing 0.55 mM $\mathbf{V_6O_{13}}^{-2}$, 0.27 mM $\mathbf{V_6O_{11}(OH)_2}^{-2}$, 0.1 M [^{n}Bu$_4$N][PF$_6$] as supporting electrolyte, and 0.05 M buffer of 1:1 N,N-Dimethylaniline/N,N-Dimethylanilinium tetrafluoroborate. The open circuit potential was allowed to equilibrate for 5 min, or until the potential was changing at a rate less than 0.005 mV/s. A 0.1 mL aliquot of a stock solution of cluster (2.5 mM stock solution) was titrated into the sample and the open circuit potential was again measured. This process was repeated 12 times, measuring a range of ratios of reduced to oxidized cluster (e.g., 0.75:1 to 1.25:1, respectively). At completion of the open circuit potential experiments, a sample of ferrocene was added and an additional cyclic voltammogram was collected in order to properly reference to the $Fc^{+/0}$ couple.

General Procedure for Confirming BDFE(O-H) of $\mathbf{V_6O_{11}(OH)_2}$ through Equilibrium Experiments. In an air free glove box, 1 mL of a 2.209 mM stock solution of $\mathbf{V_6O_{11}(OH)_2}^{-2}$ in acetonitrile and 2 mL of acetonitrile are added to a long-necked UV-Vis cuvette. The cuvette is capped with a rubber septum and removed from the glove box, where a control electronic absorption spectrum is collected. In a separate UV-Vis cuvette, 1 mL of the $\mathbf{V_6O_{11}(OH)_2}^{-2}$ stock solution is added, along with 1.5 mL of acetonitrile, along with 0.5 mL of a stock solution containing 8.838 mM TEMPO in acetonitrile. The sample was shaken several times to ensure homogeneity and allowed to sit at 25 °C for 30 minutes, whereupon an electronic absorption spectrum was collected. The extent of the reaction is determined through the absorbance measured at 750 nm, where the IVCT band belonging to $\mathbf{V_6O_{11}(OH)_2}^{-2}$ allows for concentration of each cluster to be calculated. The concentration of TEMPO and TEMPOH are established by relative amounts of reduced and oxidized cluster present in solution

Calculating the BDFE(O-H) of the reduced cluster in solution can then be performed through methods adapted from the Mayer group,[3] using Eqn. S1.

$$BDFE(O-H)_{eq} = BDFE(XH_n) - \frac{2.303RT}{n}\log\left(\frac{[XH_n]}{[X]}\right) \qquad \text{Eqn. S1}$$

Where $BDFE(O\text{-}H)_{eq}$ is the BDFE of the cluster based on where the equilibrium of the system lies, $BDFE(XH_n)$ is the reported BDFE(O-H) of the reduced version of TEMPO (66 kcal mol^{-1} in acetonitrile[2]), R is the gas constant, T is the temperature, n is the number of H-atoms transferred to one equivalent of TEMPO (n = 1 in these experiments, as TEMPO can only accept one H-atom equivalent), and $[XH_n]$ and $[X]$ are the measured concentrations of reduced and oxidized versions of the respective substrate in solution.

General Procedure for Measuring Kinetics of PCET at POV Clusters Through Electronic Absorption. Each sample was monitored using electronic absorption spectroscopy, where the absorbance at 750 nm is used to determine the concentration of the reduced cluster, $\mathbf{V_6O_{11}(OH)_2}^{-2}$, present in solution. All experiments are performed in 3 mL of acetonitrile at the desired temperature.

An initial sample of either $\mathbf{V_6O_{13}^{-2}}$ or $\mathbf{V_6O_{11}(OH)_2^{-2}}$ was prepared in acetonitrile in a air free quartz cuvette. A sample of stock solution containing the organic substrate was collected in an air-free syringe and both cuvette and syringe are removed from the glove box. The cuvette is inserted into the UV-Vis spectrophotometer and allowed to equilibrate to the desired temperature. Data collection begins as soon as the organic substrate is rapidly injected into the cuvette. Once the sample reaches equilibrium, and the absorbance of the sample is no longer changing, the data collection is stopped. The sample is then disposed of in the waste, and the cuvette is washed and dried in an oven at 125 °C for 2 hours before being brought back into the glove box.

Obtaining the best fit for the experimental results is achieved by assuming the change in absorbance while in pseudo-first order reaction conditions obey Eqn. S2.

$$A_t = A_{inf} + (A_0 - A_{inf})e^{(-kobs*t)} \qquad \text{Eqn. S2}$$

Where A_t is absorbance at time = t, A_{inf} is the absorbance of the sample once equilibrium is reached, A_0 is the initial absorbance of the sample, k_{obs} is the observed rate constant in pseudo-first order reaction conditions, and t is time in seconds. The model is fit to the experimental data using the residual sum of squares.

Method for determining k_{PCET} for the reaction between $V_6O_{11}(OH)_2^{-2}$ and anthracene. Each sample was monitored using ^{1}H NMR spectroscopy, where the diamagnetic peak belonging to the oxidized cluster, $\mathbf{V_6O_{13}^{-2}}$, allows for the extent of the reaction to be monitored over time. The rate of reaction was monitored in pseudo-first order reaction conditions, where the concentration of anthracene was held in at least 10-fold excess relative to the concentration of the cluster. The kinetic experiments were repeated using a range of concentrations of anthracene in order to obtain the k_{PCET} for the reaction. All experiments were performed at 25 °C in 0.4 mL of CD_3CN in an air-free J-Young tube.

To a J-Young tube, a 50 μL aliquot of a stock solution containing 6 mM $\mathbf{V_6O_{13}^{-2}}$ was added, along with an aliquot of a stock solution containing 20.6 mM of anthracene and 10 μL aliquot of a stock solution containing 80 mM of the internal standard, hexamethyldisiloxane (HMDS). Extra CD_3CN was added to the sample to reach a final volume of 0.4 mL. The J-Young tube was removed from the glove box and an initial ^{1}H NMR spectrum was collected, after which the sample was placed into a water bath set to 25 °C. ^{1}H NMR spectra were collected periodically over several days in order to obtain a series of spectra tracking the change in concentration of the cluster over time. The precise concentration of the cluster was determined by creating a calibration curve, where a series of ^{1}H NMR spectra were collected with various concentrations of $\mathbf{V_6O_{13}^{-2}}$ while in the presence of 2 mM of the internal standard, HMDS. This curve was then used to find [$\mathbf{V_6O_{13}^{-2}}$] at each time point by measuring the relative peak integrations between the cluster and HMDS.

Upon completion of the kinetic experiments, k_{pbs} was plotted against the concentration of anthracene initially present in solution, however, in contrast to the other kinetic experiments, this plot did not produce a linear trend with a y-intercept of approximately zero. To find the order with respect to anthracene and k_{PCET}, the natural log of the concentration of anthracene was plotted against the natural log of k_{obs}. From this plot, we are able to find both the order with respect to anthracene, and k_{PCET} according to Eqn S3-S6. In Eqn S3, we can see the proposed overall rate expression. In pseudo-first order reaction conditions we are able to simplify the rate expression to Eqn. S4, where k_{obs} can be found by Eqn. S5. Plotting the natural log of k_{obs} against the natural log of the concentration of anthracene results in Eqn. S6, where the slope of the plot indicates the order of the reaction with respect to anthracene, in this case the value is 2. The 8 accounts for the statistical correction due to the fact that $\mathbf{V_6O_{11}(OH)_2^{-2}}$ has two equivalent

O-H ligands, and two anthracene molecules are present at the rate limiting step, each with two identical C atoms that act as the active site for PCET. From the Y-intercept, we are able to find the k_{PCET} as 4.0 (± 2.7) x 10^{-4} M^{-2} s^{-1}.

$$\frac{d[\boldsymbol{V_6O_{13}^{-2}}]}{dt} = k_{PCET}[\boldsymbol{V_6O_{11}(OH)_{11}^{-2}}]^1[Anthracene]^x \quad \text{Eqn. S3}$$

$$\frac{d[\boldsymbol{V_6O_{13}^{-2}}]}{dt} = k_{obs}[\boldsymbol{V_6O_{11}(OH)_{11}^{-2}}]^1 \quad \text{Eqn. S4}$$

$$k_{obs} = 8k_{PCET}[Anthracene]^x \quad \text{Eqn. S5}$$

$$\ln\,(k_{obs}) = \ln\,(8) + \ln(k_{PCET}) + Xln[Anthracene] \quad \text{Eqn. S6}$$

Table S1. List of organic acids used in cyclic voltammetry experiments, along with the acid dissociation constants used for this study. [4-10]

Acid	Abbreviation	pK_a	pK_a Reference
Pyrazolium tetrafluoroborate	$PyrazH^+$	9.1	4
2-Chloropyridinium tetrafluoroborate	2-Cl-$PyrdH^+$	9.56	4
2-Methoxypyridinium tetrafluoroborate	2-MeO-$PyrdH^+$	9.94	4
Methyldiphenylphosphonium tetrafluoroborate	$MePh_2PH^+$	9.97	4
Anilinium tetrafluoroborate	$AnilineH^+$	10.64	4
N,N-Dimethylanilinium tetrafluoroborate	N,N-$DMAH^+$	11.47	4
Pyridinium tetrafluoroborate	$PyrdH^+$	12.53	4
2-Methylpyridinium tetrafluoroborate	2-CH_3-$PyrdH^+$	13.38	4
2-Aminopyridinium tetrafluoroborate	2-NH_2-$PyrdH^+$	14.5	4
Trimethylphosphonium tetrafluoroborate	Me_3PH^+	15.48	4
Trimethylammonium chloride	Me_3NH^+	17.61	4
Cyclohexylammonium tetrafluoroborate	$(C_6H_{11})NH_3^+$	18.36	4
Triethylammonium tetrafluoroborate	$TEAH^+$	18.83	4
Dimethylammonium chloride	$Me_2NH_2^+$	19.03	4
Thiophenol	PhSH	20.91	5, 6
4-Methoxythiophenol	4-MeO-PhSH	21.7	5, 6
1,1,3,3-Tetramethylguanidinium tetrafluoroborate	$TMGH^+$	23.35	4
DBU	$DBUH^+$	24.31	4
Phenol	PhOH	29.14	7
Diphenylamine	Ph_2NH	34.3	6, 8
Methanol	MeOH	37.44	6, 9
Acetonitrile	MeCN	39.5	6, 10

Cyclic Voltammograms. The addition of acid to $\mathbf{V_6O_{13}}^{-2}$ was done by preparing a stock solution containing the acid of interest in acetonitrile and adding in the appropriate amount in order to achieve a 2:1 ratio of acid to cluster. In order to approximate the standard reduction potentials, the point of inflection of each event was used. To determine this point, the first derivative was found of the cyclic voltammogram which can be seen as the dotted line in each figure. The point of inflection can be found as the peak located at each event, marked by a red star. In each sample, the potential is referenced to the ferrocene couple by adding in a small amount at the conclusion of the experiment and collecting an additional CV.

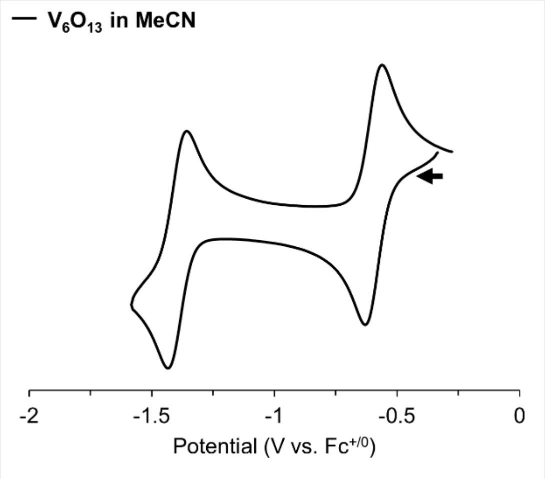

Figure S1. Cyclic voltammogram of 1 mM $\mathbf{V_6O_{13}}^{-2}$ and 0.1 M [^{n}Bu$_4$N][PF$_6$] in acetonitrile at 200 mV/s.

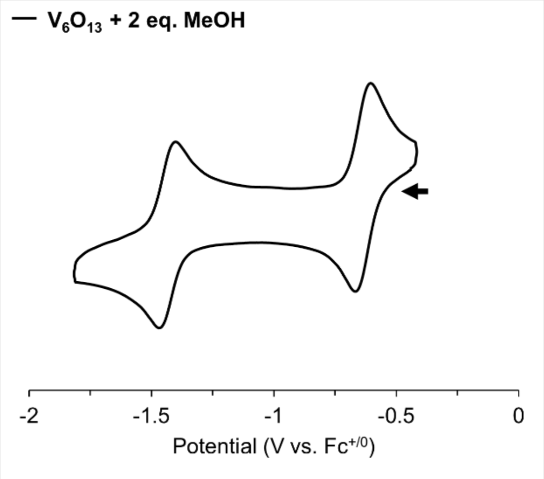

Figure S2. Cyclic voltammogram of 1 mM $\mathbf{V_6O_{13}}^{-2}$, 2 mM methanol (pK_a = 37.4), and 0.1 M [^{n}Bu$_4$N][PF$_6$] in acetonitrile at 200 mV/s.

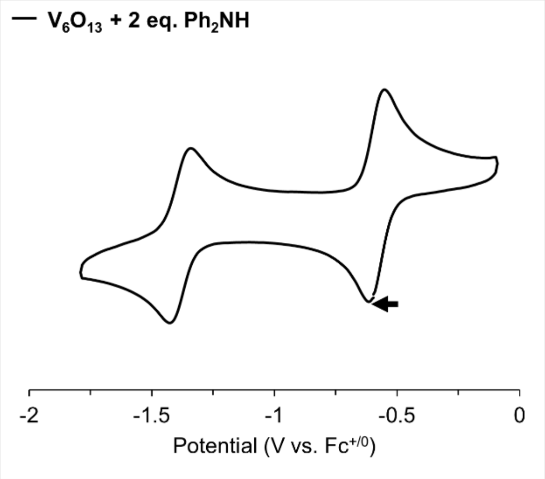

Figure S3. Cyclic voltammogram of 1 mM $\mathbf{V_6O_{13}}^{-2}$, 2 mM diphenylamine (pK_a = 34.3) , and 0.1 M [^{n}Bu$_4$N][PF$_6$] in acetonitrile at 200 mV/s .

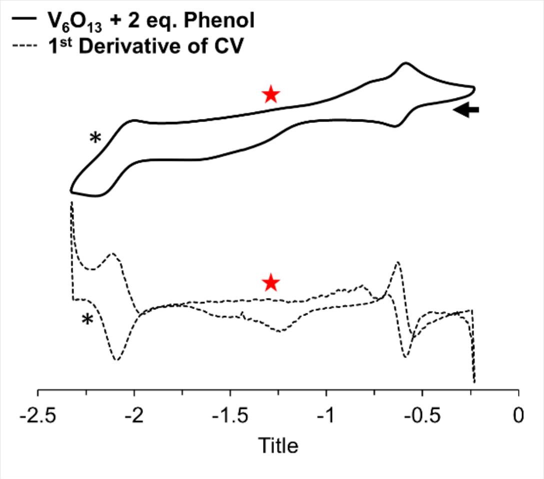

Figure S4. Cyclic voltammogram of 1 mM $\mathbf{V_6O_{13}}^{-2}$, 2 mM Phenol (pK_a = 29.2), and 0.1 M [^{n}Bu$_4$N][PF$_6$] in acetonitrile at 200 mV/s. The black star indicates the reduction of Phenol.

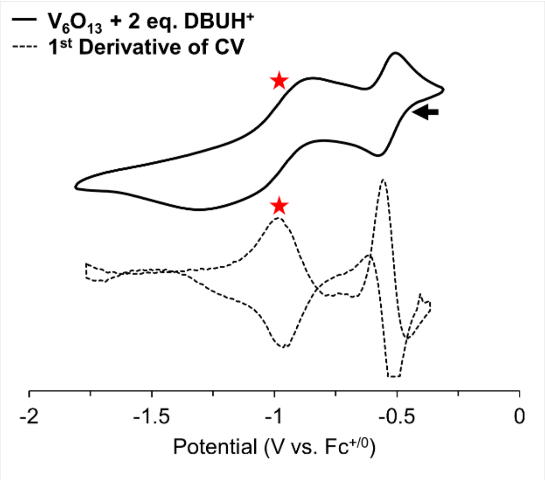

Figure S5. Cyclic voltammogram of 1 mM $\mathbf{V_6O_{13}}^{-2}$, 2 mM acidified 1,8-Diazabicyclo(5.4.0)undec-7-ene tetrafluoroborate (pK_a = 24.31), and 0.1 M [^{n}Bu$_4$N][PF$_6$] in acetonitrile at 200 mV/s.

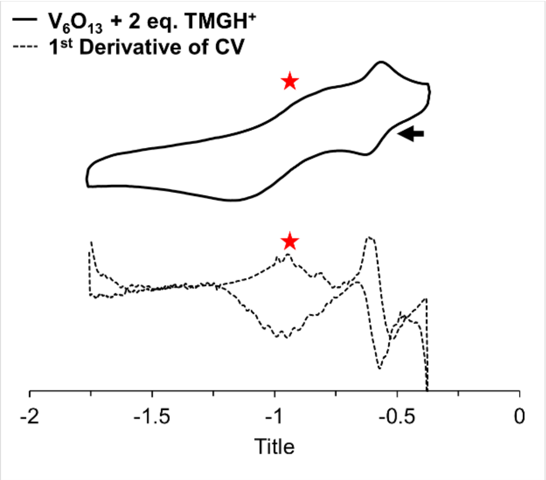

Figure S6. Cyclic voltammogram of 1 mM $\mathbf{V_6O_{13}}^{-2}$, 2 mM 1,1,3,3-tetramethylguanidinium tetrafluoroborate (pK_a = 23.35), and 0.1 M [^{n}Bu$_4$N][PF$_6$] in acetonitrile at 200 mV/s.

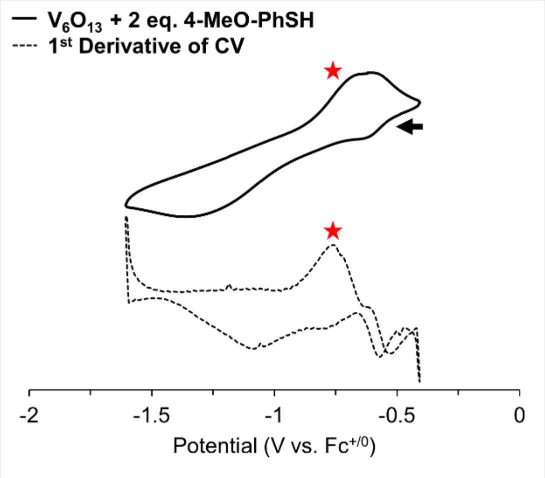

Figure S7. Cyclic voltammogram of 1 mM $\mathbf{V_6O_{13}}^{-2}$, 2 mM 4-Methoxythiophenol (pK_a = 22.6), and 0.1 M [^{n}Bu$_4$N][PF$_6$] in acetonitrile at 200 mV/s.

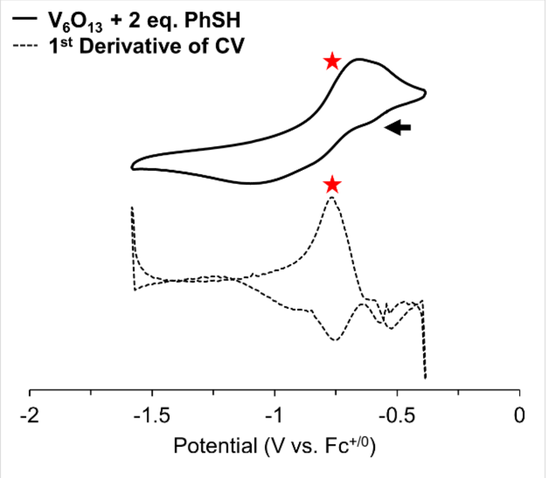

Figure S8. Cyclic voltammogram of 1 mM $\mathbf{V_6O_{13}}^{-2}$, 2 mM Thiophenol (pK_a = 21.7), and 0.1 M [^{n}Bu$_4$N][PF$_6$] in acetonitrile at 200 mV/s.

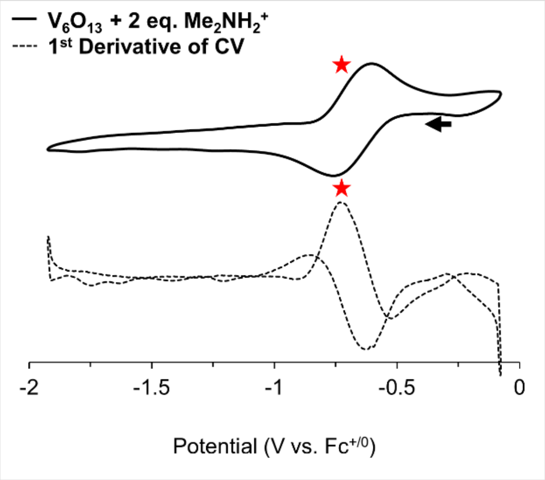

Figure S9. Cyclic voltammogram of 1 mM $\mathbf{V_6O_{13}}^{-2}$, 2 mM Dimethylammonium chloride (pK_a = 19.03), and 0.1 M [^{n}Bu$_4$N][PF$_6$] in acetonitrile at 200 mV/s.

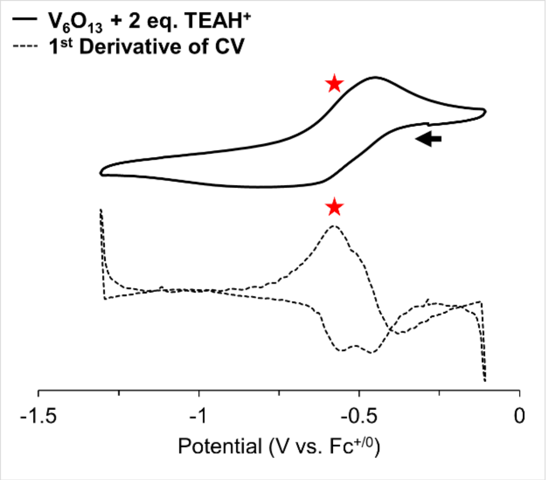

Figure S10. Cyclic voltammogram of 1 mM $\mathbf{V_6O_{13}}^{-2}$, 2 mM Triethylammonium tetrafluoroborate (pK_a = 18.83), and 0.1 M [^{n}Bu$_4$N][PF$_6$] in acetonitrile at 200 mV/s.

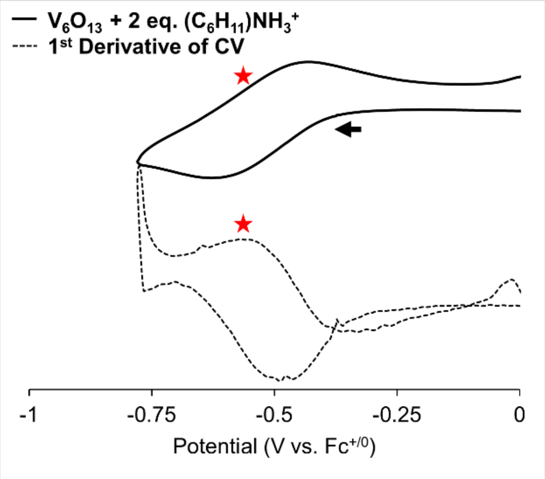

Figure S11. Cyclic voltammogram of 1 mM $\mathbf{V_6O_{13}}^{-2}$, 2 mM Cyclohexylammonium tetrafluoroborate (pK_a = 13.36), and 0.1 M [^{n}Bu$_4$N][PF$_6$] in acetonitrile at 200 mV/s.

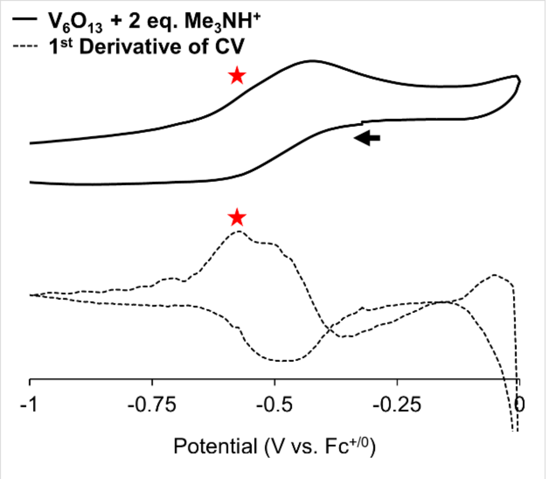

Figure S12. Cyclic voltammogram of 1 mM $\mathbf{V_6O_{13}}^{-2}$, 2 mM Trimethylammonium tetrafluoroborate (pK_a = 17.61), and 0.1 M [^{n}Bu$_4$N][PF$_6$] in acetonitrile at 200 mV/s.

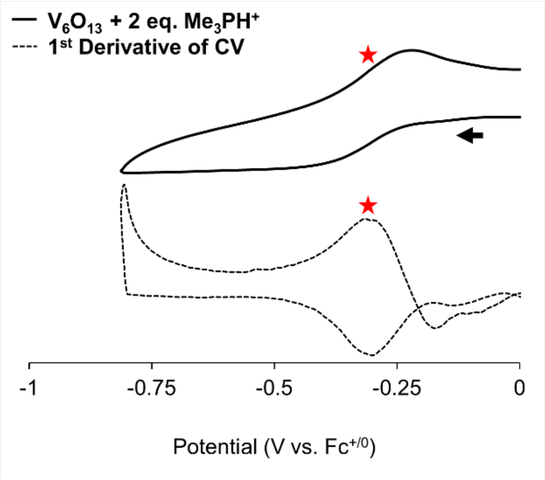

Figure S13. Cyclic voltammogram of 1 mM $\mathbf{V_6O_{13}}^{-2}$, 2 mM Trimethylphosphonium tetrafluoroborate (pK_a = 15.48), and 0.1 M [^{n}Bu$_4$N][PF$_6$] in acetonitrile at 200 mV/s.

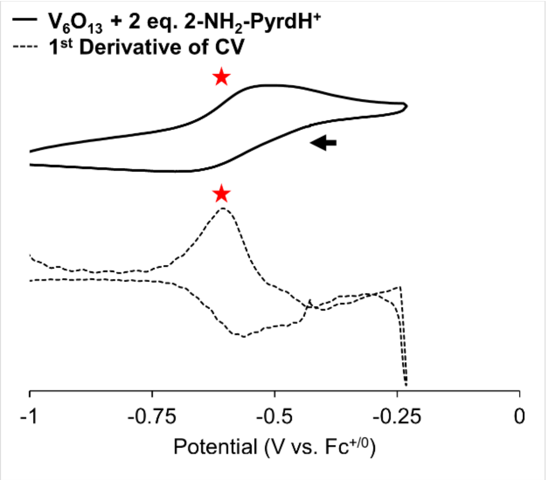

Figure S14. Cyclic voltammogram of 1 mM $\mathbf{V_6O_{13}}^{-2}$, 2 mM 2-Aminopyridinium tetrafluoroborate (pK_a = 14.5), and 0.1 M [^{n}Bu$_4$N][PF$_6$] in acetonitrile at 200 mV/s.

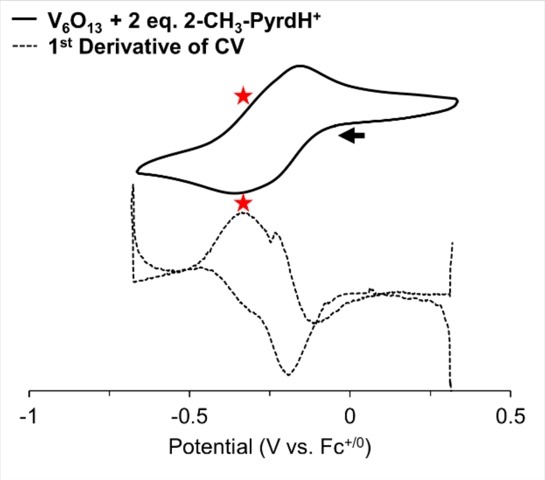

Figure S15. Cyclic voltammogram of 1 mM $\mathbf{V_6O_{13}}^{-2}$, 2 mM 2-Methylpyridinium tetrafluoroborate (pK_a = 13.28), and 0.1 M [^{n}Bu$_4$N][PF$_6$] in acetonitrile at 200 mV/s.

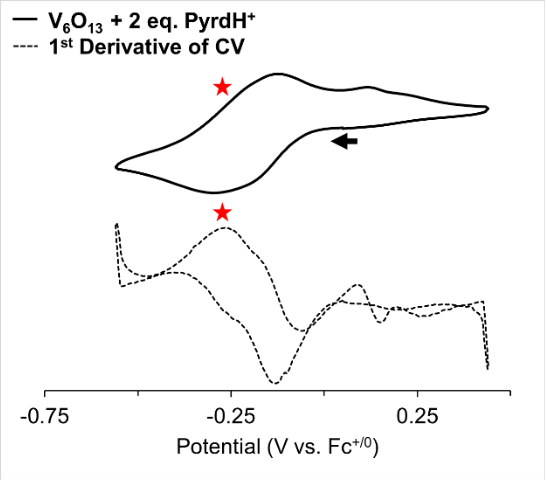

Figure S16. Cyclic voltammogram of 1 mM $\mathbf{V_6O_{13}}^{-2}$, 2 mM Pyridinium tetrafluoroborate (pK_a = 12.53), and 0.1 M [^{n}Bu$_4$N][PF$_6$] in acetonitrile at 200 mV/s.

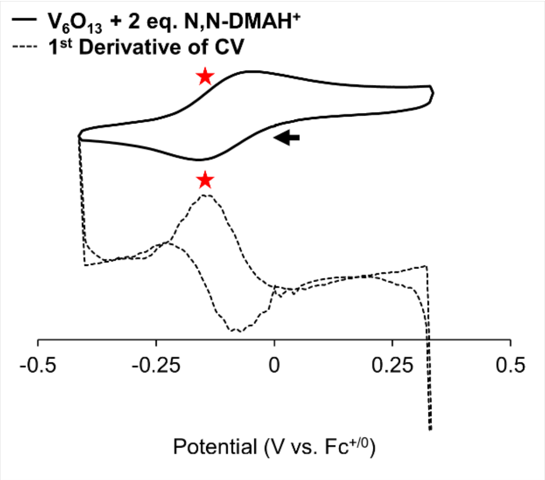

Figure S17. Cyclic voltammogram of 1 mM $\mathbf{V_6O_{13}}^{-2}$, 2 mM N,N-Dimethylanilinium tetrafluoroborate (pK_a = 11.47), and 0.1 M [^{n}Bu$_4$N][PF$_6$] in acetonitrile at 200 mV/s.

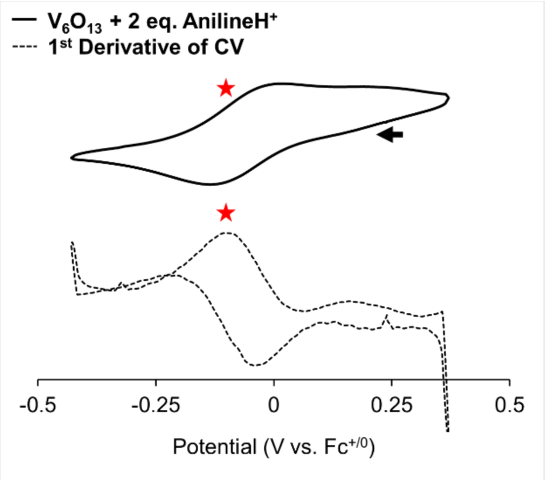

Figure S18. Cyclic voltammogram of 1 mM $\mathbf{V_6O_{13}}^{-2}$, 2 mM Anilinium tetrafluoroborate (pK_a = 10.64), and 0.1 M [^{n}Bu$_4$N][PF$_6$] in acetonitrile at 200 mV/s.

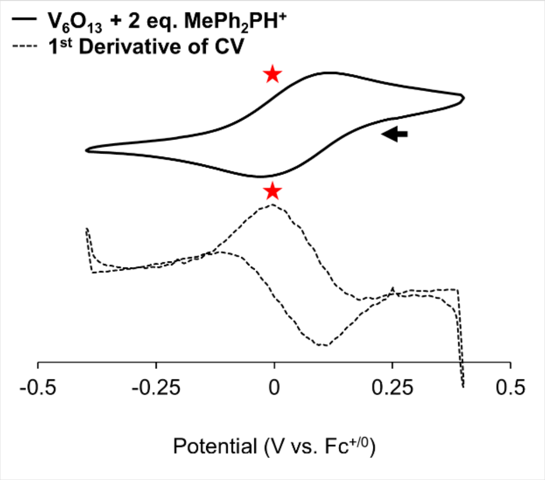

Figure S19. Cyclic voltammogram of 1 mM $\mathbf{V_6O_{13}}^{-2}$, 2 mM Methyldiphenyl-phosphonium tetrafluoroborate (pK_a = 9.97), and 0.1 M [^{n}Bu$_4$N][PF$_6$] in acetonitrile at 200 mV/s.

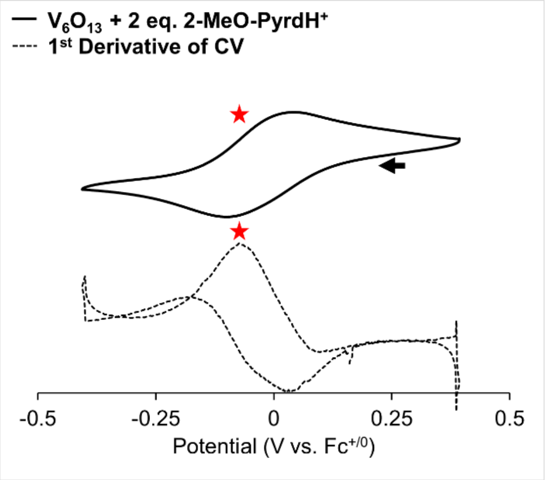

Figure S20. Cyclic voltammogram of 1 mM $\mathbf{V_6O_{13}}^{-2}$, 2 mM 2-Methoxypyridinium tetrafluoroborate (pK_a = 9.94), and 0.1 M [^{n}Bu$_4$N][PF$_6$] in acetonitrile at 200 mV/s.

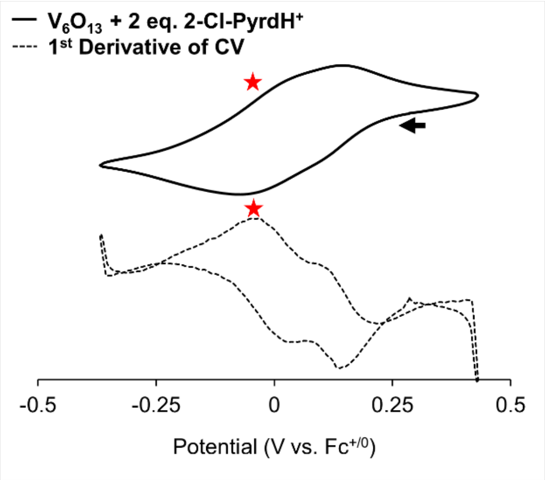

Figure S21. Cyclic voltammogram of 1 mM $\mathbf{V_6O_{13}}^{-2}$, 2 mM 2-Chloropyridinium tetrafluoroborate (pK_a = 9.56), and 0.1 M [^{n}Bu$_4$N][PF$_6$] in acetonitrile at 200 mV/s.

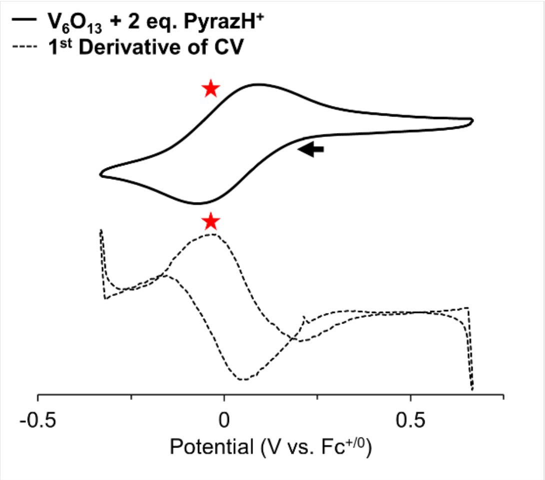

Figure S22. Cyclic voltammogram of 1 mM $\mathbf{V_6O_{13}}^{-2}$, 2 mM Pyrazolium tetrafluoroborate (pK_a = 9.1), and 0.1 M [^{n}Bu$_4$N][PF$_6$] in acetonitrile at 200 mV/s.

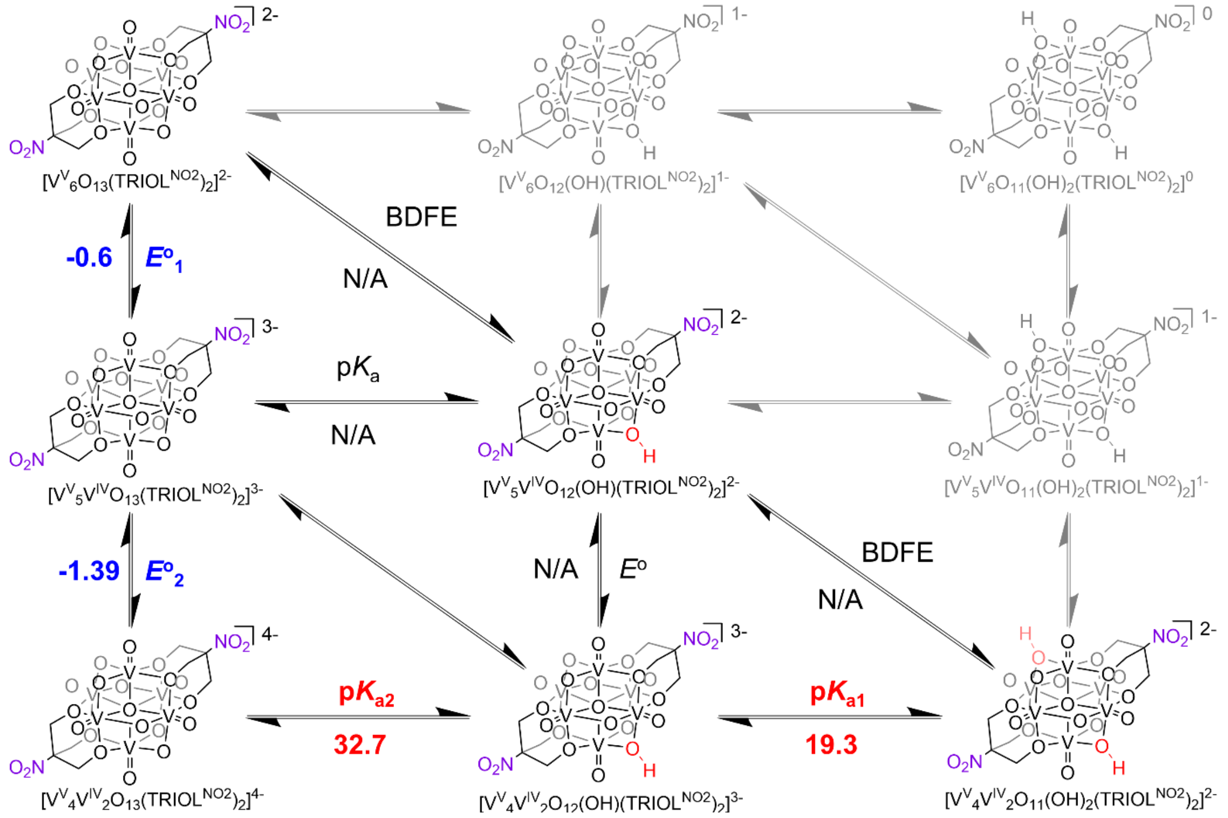

$$\mathrm{BDFE(O\text{-}H)_{avg}} = (1.37(\mathrm{p}K_{a1}+\mathrm{p}K_{a2})+23.06(E^o_1+E^o_2)+2C_g)/2$$

Scheme S.1. Full square scheme depicting each possible reaction pathway for the formation of $\mathbf{V_6O_{11}(OH)_2}$ (bottom right) from the fully oxidized species, $\mathbf{V_6O_{13}}$ (top left). Each reduction potential and acid dissociation constant obtained from the series of cyclic voltammogram experiments (see Figure 1 and S1-S22) have been included. From these thermochemical parameters, we are able to calculate the average BDFE(O-H) of the reduced cluster in acetonitrile through the equation shown below the square scheme (C_g = 52.6 kcal mol^{-1} in acetonitrile). Of note, we are unable to identify the BDFE(O-H) of each hydroxide ligand at the reduced cluster surface due to the fact that one acid dissociation constant and reduction potential were unable to be obtained experimentally (labeled as N/A on the square scheme), as a result, we were limited to using the average of both of these events.

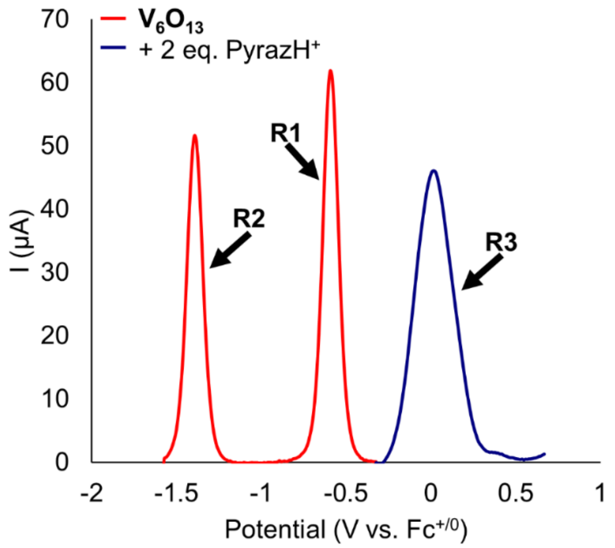

Figure S23. Figure comparing the square wave voltammograms of 1 mM of isolated $\mathbf{V_6O_{13}^{-2}}$ (red) and 1 mM of $\mathbf{V_6O_{13}^{-2}}$ in the presence of 2 mM Pyrazolium tetrafluoroborate (blue). R1 and R2 each belong to a single electron reduction event at $\mathbf{V_6O_{13}^{-2}}$ in the absence of acid, while R3 is the single reduction event observed upon addition of the acid to the cluster. By comparing the relative integrations between R3 and either R1 or R2, we are able to determine the relative amount of charge passed to the cluster by the electrode. The relative integrations for R3/R1 is equal to 1.75, and the relative integrations for R3/R2 is 2.11. suggesting that two electrons are being transferred to the cluster in a single event upon the addition of sufficiently strong acid.

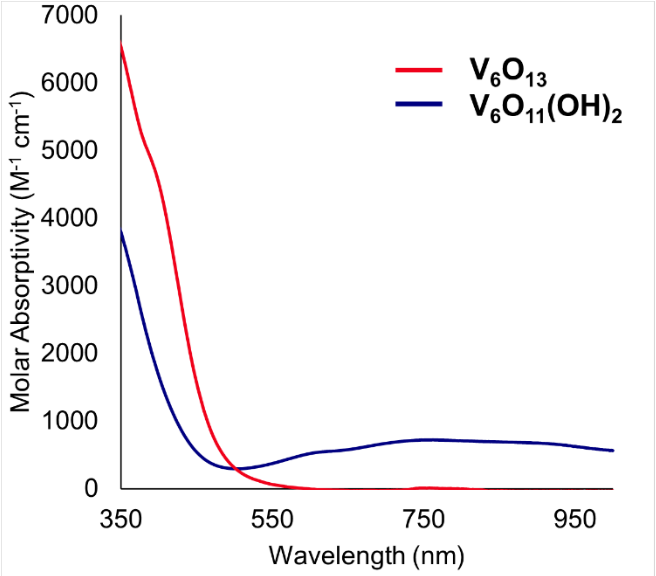

Figure S24. Electronic absorption spectra for $\mathbf{V_6O_{13}^{-2}}$ and $\mathbf{V_6O_{11}(OH)_2^{-2}}$. Both spectra are taken in acetonitrile at 25°C.

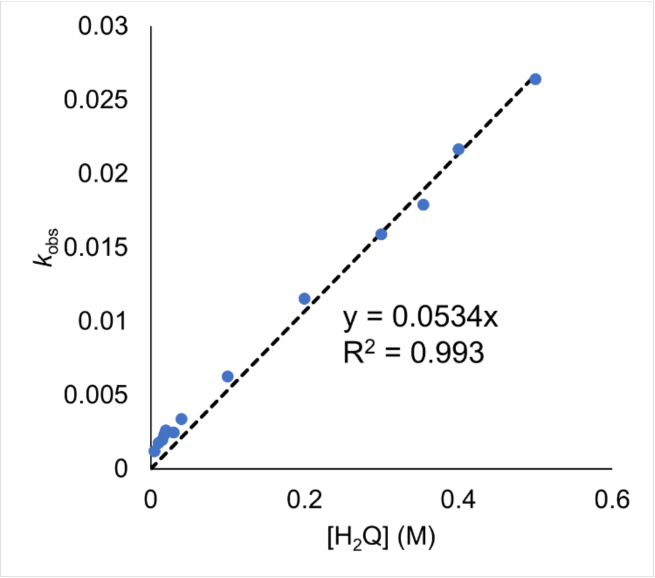

Figure S25. Plot of k_{obs} against the concentration of H_2Q initially in solution for the reaction between $\mathbf{V_6O_{13}^{-2}}$ and H_2Q. Each sample contains 0.2 mM of $\mathbf{V_6O_{13}^{-2}}$ in acetonitrile and run at 25 ºC. The linear trend of these results indicates the reaction is first order with respect to H_2Q, where the second order rate constant, k_{PCET} can be determined from the slope of the best fit line (black dotted line). k_{PCET} = $^1/_{12}$ * slope, where the $^1/_{12}$ accounts for the two chemically identical O-H ligands at H_2Q, along with the six chemically identical bridging oxide ligands of the cluster.

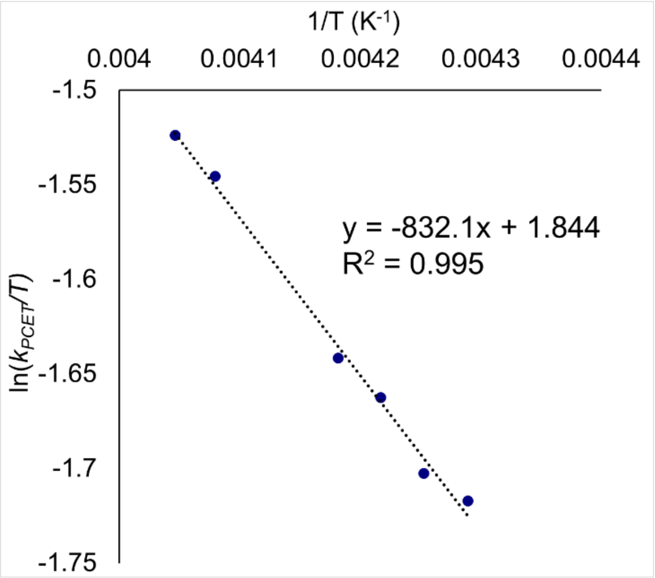

Figure S26. Eyring plot depicting the rate constant, k_{PCET}, for the reaction of $\mathbf{V_6O_{13}^{-2}}$ and iAscH$^-$ at various temperatures. Due to the rapid rate of reaction, k_{PCET} at 25 ºC was unable to be directly measured. Instead, the rate constant is extrapolated from the results shown here, where it is assumed that the linear trend is constant through 25 ºC. Each sample contains 0.25 mM $\mathbf{V_6O_{13}^{-2}}$ and 2.5 mM of iAscH$^-$ in acetonitrile.

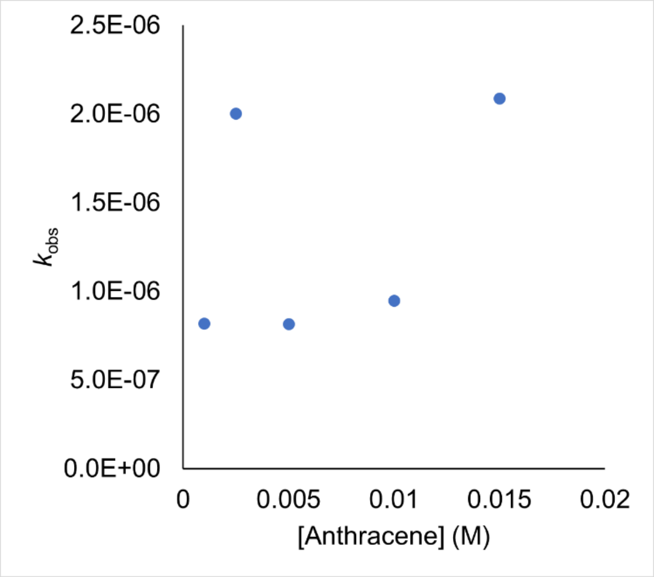

Figure S27. Plot of the observed rate constant, k_{obs}, against the concentration of anthracene initially in solution for the reaction between $\mathbf{V_6O_{11}(OH)_2^{-2}}$ and anthracene. Each experiment was run with 0.75 mM $\mathbf{V_6O_{11}(OH)_2^{-2}}$ in acetonitrile at 25 °C. The lack of trend present in the results suggests that O_2 is leaking into the sample and inducing oxidation of the cluster to.

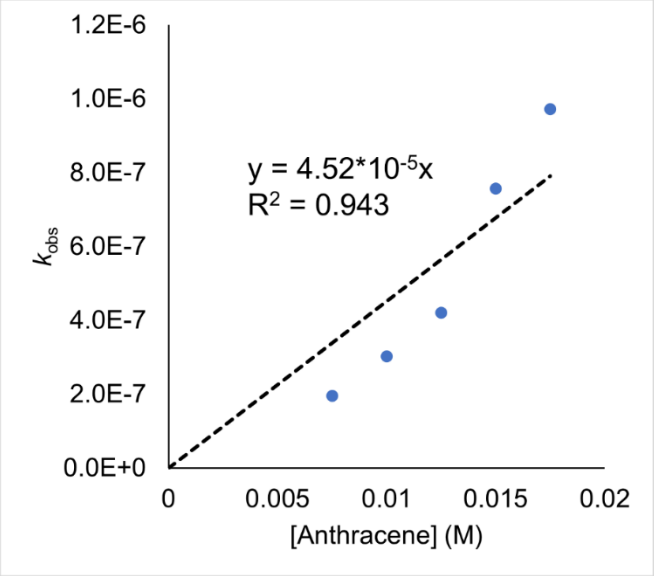

Figure S28. Plot depicting the poor fit of a linear trend line for the observed rate constant plotted against the concentration of anthracene initially present in the reaction between $\mathbf{V_6O_{11}(OH)_2^{-2}}$ and anthracene. Each experiment was monitored by ^{1}H NMR spectroscopy, with each sample containing 0.75 mM $\mathbf{V_6O_{11}(OH)_2^{-2}}$ in CD_3CN. The poor fit of the dotted black line to the experimental results suggests the order with respect to anthracene is likely not 1.

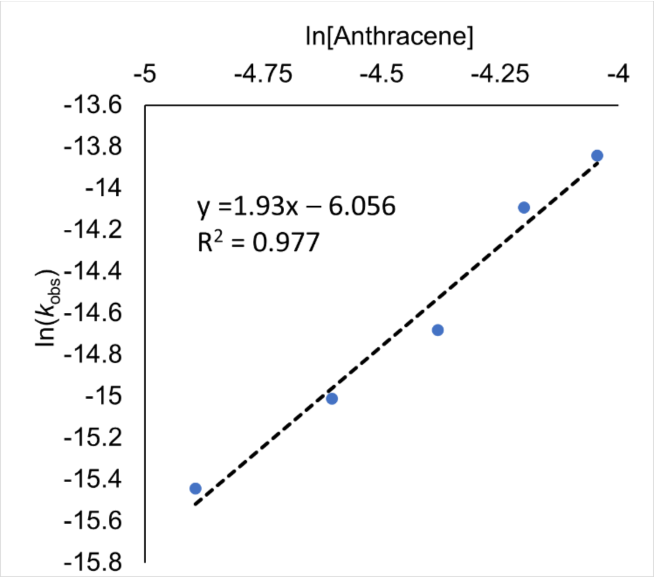

Figure S29. Plot of the natural log of k_{obs} against the natural log of the concentration of anthracene initially present at the beginning of the reaction between $\mathbf{V_6O_{11}(OH)_2^{-2}}$ and anthracene. The slope of approximately 2 suggests the order of the reaction with respect to anthracene is 2, and the overall rate constants, k_{PCET} can be determined from the y-intercept (see experimental section for more details).

References.

1. Chen, Q.; Goshorn, D. P.; Scholes, C. P.; Tan, X. L.; Zubieta, J., Coordination compounds of polyoxovanadates with a hexametalate core. Chemical and structural characterization of $[V^{V}_6O_{13}[(OCH_2)_3CR]_2]^{2-}$, $[V^{V}_6O_{11}(OH)_2[(OCH_2)_3CR]_2]$, $[V^{IV}_4V^{V}_2O_9(OH)_4[(OCH_2)_3CR]_2]^{2-}$, and $[V^{IV}_6O_7(OH)_6](OCH_2)_3CR]_2]^{2-}$. *J. Am. Chem. Soc.* **1992,** *114*, 4667-4681.
2. Wise, C. F.; Agarwal, R. G.; Mayer, J. M., Determining Proton-Coupled Standard Potentials and X–H Bond Dissociation Free Energies in Nonaqueous Solvents Using Open-Circuit Potential Measurements. *J. Am. Chem. Soc.* **2020,** *142*, 10681-10691.
3. Agarwal, R. G.; Kim, H. J.; Mayer, J. M., Nanoparticle O-H Bond Dissociation Free Energies from Equilibrium Measurements of Cerium Oxide Colloids. *J. Am. Chem. Soc.* **2021,** *143*, 2896-2907.
4. Tshepelevitsh, S.; Kütt, A.; Lõkov, M.; Kaljurand, I.; Saame, J.; Heering, A.; Plieger, P. G.; Vianello, R.; Leito, I., On the Basicity of Organic Bases in Different Media. *Eur. J. Org. Chem.* **2019**, 6735-6748.
5. Bordwell, F. G., Equilibrium acidities in dimethyl sulfoxide solution. *Acc. Chem. Res.* **1988,** *21*, 456-463.
6. Yu, H.-Z.; Yang, Y.-M.; Zhang, L.; Dang, Z.-M.; Hu, G.-H., Quantum-Chemical Predictions of pKa's of Thiols in DMSO. *J. Phys. Chem. A* **2014,** *118*, 606-622.
7. Kütt, A.; Tshepelevitsh, S.; Saame, J.; Lõkov, M.; Kaljurand, I.; Selberg, S.; Leito, I., Strengths of Acids in Acetonitrile. *Eur. J. Org. Chem.* **2021,** 1407-1419.
8. Maran, F.; Celadon, D.; Severin, M. G.; Vianello, E., Electrochemical determination of the pKa of weak acids in N,N-dimethylformamide. *J. Am. Chem. Soc.* **1991,** *113*, 9320-9329.
9. Huffman, L. M.; Casitas, A.; Font, M.; Canta, M.; Costas, M.; Ribas, X.; Stahl, S. S., Observation and Mechanistic Study of Facile C-O Bond Formation between a Well-Defined Aryl–Copper(III) Complex and Oxygen Nucleophiles. *Chem. Eur. J.* **2011,** *17*, 10643-10650.
10. Bordwell, F. G.; Branca, J. C.; Bares, J. E.; Filler, R., Enhancement of the equilibrium acidities of carbon acids by polyfluoroaryl substituents. *J. Org. Chem.* **1988,** *53*, 780-782.
